# Simultaneous Digital Design and Additive Manufacture of Structures and Materials

**DOI:** 10.1038/s41598-018-33454-3

**Published:** 2018-10-22

**Authors:** Narasimha Boddeti, Zhen Ding, Sawako Kaijima, Kurt Maute, Martin L. Dunn

**Affiliations:** 1 Digital Manufacturing and Design (DManD) Centre, Singapore University of Technology and Design, Singapore, Singapore; 2 Department of Aerospace Engineering Sciences, University of Colorado, Boulder, United States; 3 College of Engineering and Applied Sciences, University of Colorado, Denver, United States; 4 Shenzhen Key Laboratory of Nanobiomechanics, Shenzhen Institutes of Advanced Technology, Chinese Academy of Sciences, Shenzhen, China

## Abstract

The integration of emerging technologies into a complete digital thread promises to disrupt design and manufacturing workflows throughout the value chain to enable efficiency and productivity transformation, while unlocking completely new design freedom. A particularly appealing aspect involves the simultaneous design and manufacture of the macroscale structural topology and material microstructure of a product. Here we demonstrate such a workflow that digitally integrates: *design automation* – conception and automation of a design problem based on multiscale topology optimization; *material compilation* – computational geometry algorithms that create spatially-variable, physically-realizable multimaterial microstructures; and *digital fabrication* – fabrication of multiscale optimized components via voxel-based additive manufacturing with material jetting of multiple photo-curable polymers. We validate the digital design and manufacturing workflow by designing, fabricating, and testing a series of structures that illustrate capabilities, show how it empowers the exploitation of new design freedom, and even challenges traditional design principles relating form, structure, and function.

## Introduction

Physical product development and management frameworks are well established and combine creative and structured processes that involve various forms of conceptualization, design, development, manufacture, marketing, and sales. An especially important part of product development workflows centers on design and manufacturing where sophisticated computer-aided design (CAD) and computer-aided manufacturing (CAM) technologies integrate design, engineering analysis, manufacturing process planning, and fabrication. New digital technologies are promising to disrupt existing CAD-CAM workflows, better integrate skill sets and trades in the value chain, and simultaneously increase efficiency and productivity while unlocking new capabilities. A particular aspect of product development that stands to be transformed is the age-old paradigm of designing form and function in the context of specified materials that influence product performance through their particular properties, e.g., stiffness and strength. This generally proceeds in a manner that involves computer-aided design of geometry incorporating manufacturing constraints, followed by engineering analysis by the finite element method where the material behavior is considered; sophisticated approaches involve these in an iterative approach to optimize designs with respect to prescribed objective(s) and constraints. Material properties are simply abstracted representations of the effects of their microstructure, e.g., molecular structure of a polymer. An important concept in material description is that of scale where the length scale of the microstructure variation has to be adequate for the concept of an average material property over a representative volume to be useful. The ability to use these concepts to describe the complex microstructural phenomena in a form that simplifies engineering analysis and design has been particularly valuable, but at the same time has compartmentalized knowledge into specific domains, e.g., structures and materials.

New and emerging digital technologies in both design automation and manufacturing processes are challenging this paradigm. Additive manufacturing (AM) is a particularly exciting one^1,2^ with the ability to fabricate a product (a structure in a mechanical context) from nothing by continually adding material point by point until a final 3D structure is formed. Newer AM technologies, e.g., fused deposition modeling^3–7^, projection micro-stereolithography^8^, direct ink writing^9^, powder fusion via powder bed^10–12^ or direct energy deposition^13^ and by extension 4D printing^14–16^ promise this manufacturing freedom, but the most advanced is probably the voxel-based placement of polymers by material jetting^17^. Here multiple polymers ranging from stiff (Young’s modulus, E ~1 GPa) and glassy to soft (E ~1 MPa) and elastomeric can be precisely placed in voxels of dimensions of 42.3 × 84.7 × 15 μm. This results in about 18,000 independently addressed voxels in a mm^3^ volume and creates unprecedented freedom to design and manufacture. While this comes with new challenges associated with the digital representation and manipulation of both geometry and material, it creates the potential for a completely new paradigm of product development – the simultaneous design and manufacture of structure and material (in the mechanical case). This includes innovative ways to blend materials spatially and create functional property gradients – that can improve performance, optimize use of materials, and enable new and previously unobtainable functionality. It even provocatively questions the need for traditional separation of material and structure.

These powerful manufacturing technologies are outpacing the development of design methods and tools that allow the exploitation of the new freedom they provide. A connection is emerging, though, with the computational design automation approach of topology optimization^18,19^ that optimally determines where to place materials in a 3D design domain to achieve a desired function. In fact the widespread adoption of topology optimization has been hindered by the inability to physically realize optimal designs with traditional manufacturing technologies, however AM is a natural companion as it provides the freedom to exploit its capabilities.

While most approaches that connect topology optimization to additive manufacturing still separate the material and structure and create optimal structures from specified materials, efforts are emerging along two directions to simultaneously design structure and material^20–33^. The first iteratively determines macroscale topology and spatially varying microstructure by bridging the micro- and macro-scales through numerical homogenization^21,25,26,30^. This is computationally intensive and has practical limitations with respect to manufacturability centered around connecting the varying microstructure designs. The second trades design freedom for increased efficiency and manufacturability by limiting the possible microstructure designs to a pre-computed material gamut^27,32^ or just one^23,24^. Despite these emerging connections, the integration into a digital design-manufacturing workflow that exploits the potential is nascent as only one^32^ physically realizes the optimized structures.

Here we present an integrated digital design-manufacturing workflow that allows the simultaneous design of the macroscopic topology of a structure, and the microstructure and its 3D spatial variation of the materials from which it is made. We emphasize optimal mechanical performance in the context of elasticity theory, but the approach is generalizable to other physical phenomena that are described by continuum field equations. Our digital workflow consists of three elements: (i) a *design automation* process that integrates mathematical homogenization of a two-phase microstructure into a multiscale topology and microstructure design optimization formulation to determine the optimal layout of material and its microstructure in 3D; (ii) a *material compilation* process to project the mathematical description of the optimal macro and microstructure into a physically realizable 3D material layout and generate machine code for fabrication; and (iii) a *digital fabrication* step that drives precise voxel-based fabrication with multimaterial photopolymer material jetting. Thus, our *design automation* approach falls under the second of the aforementioned directions^23,24,27,32^ where we restrict ourselves to a single two-phase microstructure design. The distinctive features of our work compared to prior work in the literature^20–33^ are use of analytical homogenization to bridge scales, connection of design automation to fabrication for 2- and 3-dimensional structures through an integrated digital workflow, and the demonstration and experimental validation of the complete design-fabrication workflow.

It is well-known that the mathematically-optimal microstructure in a multiscale optimization approach is a so-called infinite-rank laminate of infinitesimal layer thickness^34–36^, but this is not physically realizable. Specific manufacturing technologies that can approximate the optimal microstructure have limitations as well; the most advanced are recent efforts to create spatially-varying lattice microstructures^32,33^ that approximate optimality, but these are problematic because support material needs to be provided and removed and this is challenging/impossible with many 3D additive manufacturing technologies. In order to overcome these challenges, we instantiate our approach in the context of a specific material microstructure – a two-phase fibrous composite with short fibers embedded in a matrix. This microstructure is well understood and widely used in various technologies, a reasonably wide range of material behavior, including anisotropy, can be achieved by varying the parameters that describe the microstructure, and the fibrous microstructure enables the use of accurate analytical homogenization which reduces computational complexity. Furthermore, the short-fiber microstructure can be physically realized with voxel-based material jetting without the need of internal support (powder, liquid, or gel) removal. We specifically employ voxel-based AM to take advantage of its exquisite ability to place arbitrary materials at the voxel level (42.3 × 84.7 × 15 μm). With this, we can create stiff inclusions of any size and shape giving us the ability to realize tailored fiber-based microstructures of spatial and material property resolution that are currently unobtainable by any other method. This specific microstructure, albeit with its limitations, provides a valuable step toward realizing the paradigm of simultaneous design and manufacture of structures and materials, and in essence, blurs the traditional boundary between material and structure.

## Results

### Digital Design and Manufacturing Workflow

**Figure 1:**
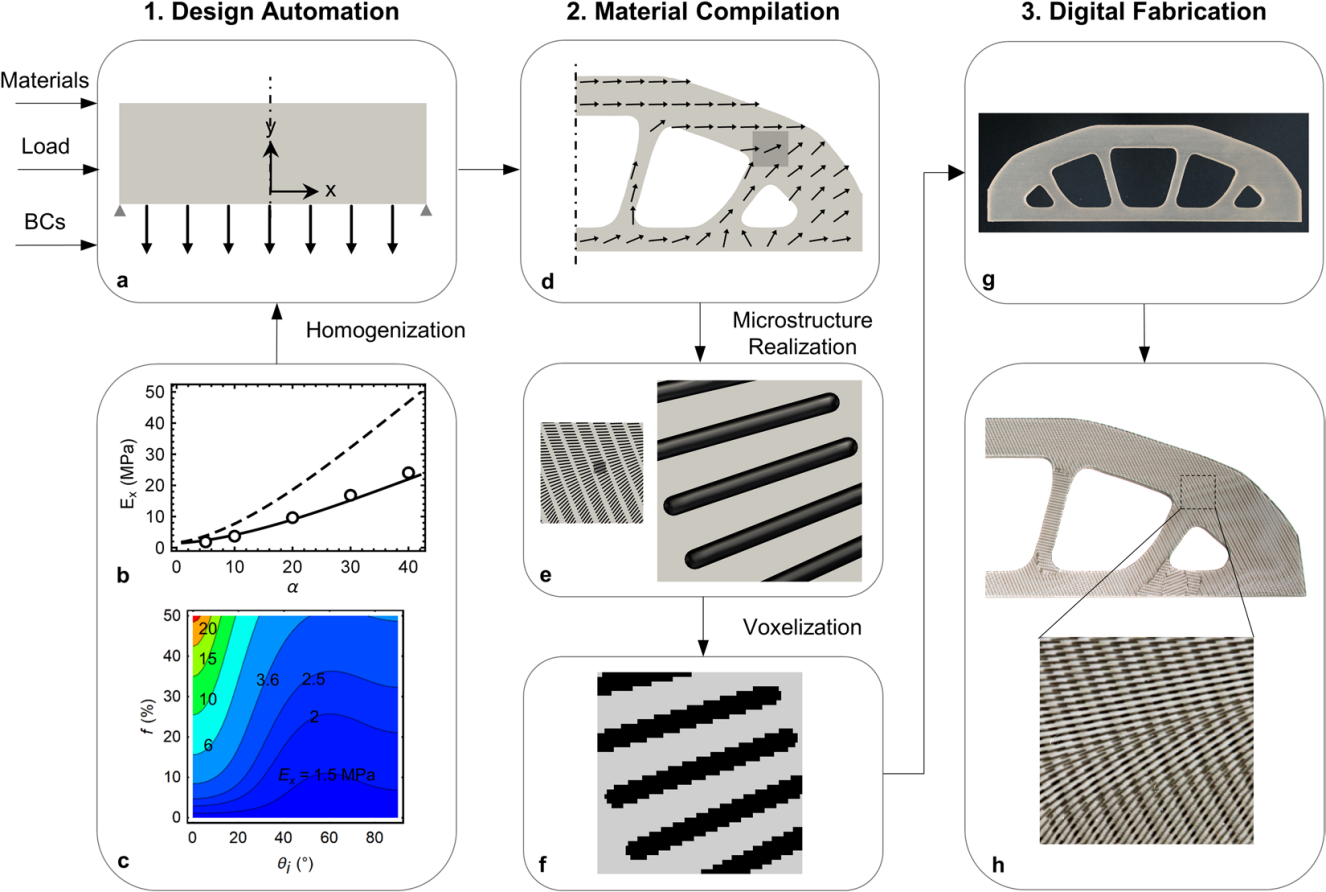
Workflow for the simultaneous digital design and manufacture of macroscopic structure topology and material microstructure – 1. Design Automation, 2. Material Compilation and 3. Digital fabrication. (**a**) Formal design problem setup showing design domain, loads, and boundary conditions. (**b**) Variation of *E*_*x*_ = (**C**^−1^)_11_^−1^ with *α* with other microstructural parameters fixed (*f* = 0.1 and 0.2 for solid and dashed curves respectively and *θ*_*i*_ = *θ*_*o*_ = 0): theory (curves) and measurements (symbols). (**c**) Variation of *E*_*x*_ = (**C**^−1^)_11_^−1^ with *f* and *θ*_*i*_ while other parameters are fixed (*α* = 10 and *θ*_*o*_ = 0). (**d**) Multi-scale simultaneous optimization for macroscale topology and microstructure (fiber orientation indicated by arrows). (**e**) Microstructure geometry realized for the shaded region shown in (**d**). (**f**) Voxelization for the zoomed out sub-region shown in (**e**) with black and gray colors representing fiber and matrix respectively. Physical structure printed with: (**g**) actual soft (matrix) and stiff (fiber) materials used in simulations; and (**h**) clear (matrix) and black (fiber) colored materials with just two layers of fibers for visualization.

Our integrated digital workflow for the simultaneous design and manufacture of the macroscopic topology of a structure and the microstructure of the materials from which it is made is shown in Fig. 1. The workflow consists of three main steps – *design automation*, *material compilation*, and *digital fabrication*; each of these consist a set of, mostly digital, sub-processes that are implemented into a software system and are described in detail in the Supplementary Information (SI). The detailed representations (Figs S1–8) admit digital and analog entities as will likely always be necessary, and provide a simple structure to enable digitization strategies, upon which entities can be disassembled, reconfigured, etc. to create new workflows and enable rapid product development.

The workflow begins with aggregation of physical performance specifications, raw material specifications, and manufacturing constraints into a design problem formulated as a mathematical optimization problem with an objective and constraints. Concurrently, we specify a material microstructure (here a two-phase short-fiber composite) and design parameters associated with it. If desired, we can replace the composite microstructure with another microstructure, such as a random dithering of voxels or a lattice. To design the structure we consider a 3D design domain, Ω, consisting of an anisotropic elastic continuum with properties that vary spatially in 3D with prescribed loads and boundary conditions (Fig. 1a). At any material point ***x***(*x*, *y*, *z*) (***x* ∈ **Ω), the elastic stiffness is the symmetric 4^th^ order tensor **C**(***x***) expressed in a global coordinate system. **C**(***x***) derives from the fact that the material microstructure varies with position throughout the solid; we assume that each material point ***x*** consists of a short-fiber composite microstructure that can be homogenized and its elastic behavior represented by **C**(***x***). To this end we adopt a mathematical homogenization approach, subject to the usual restrictions on the size of the required representative volume element at ***x***, to describe **C**(***x***) in terms of the underlying microstructure which is parameterized by a fiber aspect ratio (length/diameter) *α* and volume fraction *f*, as well as the prescribed isotropic stiffness tensors of each phase (matrix **C**_m_ and fibers **C**_f_). For simplicity, we adopt the well-known Mori-Tanaka homogenization approach^37^ which when **C**_m_ and **C**_f_ are specified delivers **C**_**l**_(***x***) = **C**_**l**_(*α*(***x***), *f*(***x***)) in terms of a principal material coordinate system aligned with the local fiber direction. The stiffness in global coordinates **C**, via standard tensor transformation of **C**_**l**_, is then **C**(***x***) = **C**(*α*(***x***), *f*(***x***), *θ*_*i*_(***x***), *θ*_*o*_(***x***)) where *θ*_*i*_(***x***) and *θ*_*o*_(***x***) are the rotation angles used to transform the fiber orientation from a local to global coordinate system (see Section 2.1, SI).

Figure 1b and c show the influence of the microstructure (volume fraction, aspect ratio, and fiber orientation) on the elastic stiffness of the composite along *x*-axis, *E*_*x*_ = (**C**^−1^)_11_^−1^ and other components follow relationships given by the Mori-Tanaka theory^37–41^. In order to validate the homogenization approach we fabricated a series of aligned short fiber composite samples with varying *α* and measured their elastic moduli; Fig. 1b shows that theory agrees well with experimental results (open circles; see Section 5, SI for details). Here and in later calculations, we used values of 1.2 MPa and 1.03 GPa for the isotropic elastic moduli of the matrix and fiber respectively, obtained from our measurements, while we assumed 0.4 for the Poisson’s ratio of both the matrix and fibers.

We formulate the engineering design problem as a topology optimization problem where we represent performance targets such as stiffness, weight, a target position etc., in an objective function, *z* that is minimized. Similarly, we represent design and manufacturing restrictions (e.g., limit on volume or mass usage, maximum stress, etc.) in either inequality or equality constraints (denoted by *g* and *h* respectively). Formally, the optimization problem is:

1 $$\begin{array}{cc}\mathop{min}\limits_{s} & z({\boldsymbol{u}}({\boldsymbol{s}}),\,{\boldsymbol{s}})\\ {\rm{s}}{\rm{u}}{\rm{b}}{\rm{j}}{\rm{e}}{\rm{c}}{\rm{t}}\,{\rm{t}}{\rm{o}} & {g}_{i}({\boldsymbol{u}}({\boldsymbol{s}}),\,{\boldsymbol{s}})\le 0\,\,i=1,\,\ldots ,\,m\\  & {h}_{j}({\boldsymbol{u}}({\boldsymbol{s}}),\,{\boldsymbol{s}})=0\,\,j=1,\,\ldots ,\,n\\  & {{\boldsymbol{s}}}_{l}\le {\boldsymbol{s}}\le {{\boldsymbol{s}}}_{u}\end{array}$$

Here, the objective and constraints are functions of optimization variables ***s*** and the displacement field ***u***, which comes from the finite element solution of the equations of elastostatics: ∇**σ**(***u***) + ***b* = 0** where **σ** and ***b*** are the stress tensor and the body force vector respectively. The stress **σ** and strain **ε** are related by Hooke’s law for an anisotropic solid, **σ** = **C**(***s***) **ε**(***u***). Later we specialize eq. (1) to specific problem formulations.

To solve the optimization problem in eq. (1) we adopt the density-based SIMP^18^ (Solid Isotropic Material with Penalization) topology optimization approach, wherein the material stiffness tensor is interpolated by a normalized density distribution, *ρ*(***x***) (0 ≤ *ρ* ≤ 1) with *ρ* = 1 implying existence of material and *ρ* = 0 that of non-existence i.e., void. The density distribution then defines material layout and thus the topology. This means, **C**(***x***)** = ***ρ *^*p*^**C**(*α*, *f*, *θ*_*i*_, *θ*_*o*_). Here *p* (typically = 3) is the SIMP exponent that in maximal stiffness (or similar) problems serves to penalize intermediate densities (0 < *ρ* < 1) and push optimal designs to exhibit distinct separation of void and material. The demarcation between void and material is specified by an isocontour of *ρ* = *ρ*_*th*_.

In our design problem formulations, we can take any combination of *ρ*, *α, f, θ*_*i*_ and *θ*_*o*_ as design variables. As such, *ρ* defines the macroscale topology and a combination of *α, f, θ*_*i*_, and *θ*_*o*_ specifies the microstructure, thus enabling multiscale topology optimization. The objective and constraints are usually functions of these design variables which are all in turn defined as linear functions of the regularized optimization variables, ***s*** (see Section 2.2, SI). We calculate gradients of the objective and constraints with respect to the ***s*** (see Section 2.3, SI), and solve the optimization problem numerically until convergence.

Figure 1d shows the optimal result of the design automation problem in Fig. 1a where structural stiffness is maximized with fixed *α, f* and *θ*_*o*_. The boundary of the macro-scale topology results from an isosurface of *ρ*_*th*_ and the microstructure is illustrated by arrows aligned along the optimal fiber orientations, *θ*_*i*_.

The design automation approach provides as output a parameterized representation of the underlying microstructure (*α, f, θ*_*i*_*, θ*_*o*_) that determines the anisotropic stiffness tensor as a function of position. A *material compiler* then projects this to an explicit geometrical realization that can be manufactured (details in Fig. S4, SI). The short fiber microstructure has the benefit of filling the entirety of the optimal structure thus defining the macroscale topology and also ensuring material and geometrical continuity at the microscale which is a challenge with discontinuous microstructures like lattices. We represent the short fibers by cylinders of radius, *r*_*f*_ with hemispherical end caps and fiber length *l*_*f*_ = *2 α r*_*f*_ (Fig. S5, SI). We specified *r*_*f*_ to be large enough so that based on the resolution of the 3D printer, a sufficient number of voxels make up the fiber and it behaves as described by elasticity theory, but as small as possible so that adequate scale separation between a fiber aggregate at a material point and features at the structure scale exists to render the homogenization concept applicable (in our case 2*r*_*f*_ = 0.36 mm as described later). We parameterize the fiber distribution with a spatially-variable hexagonal packing and adjust the two fiber spacing parameters (lateral and axial distances between the centroids of neighboring fibers; Fig. S5 and Section 3.1, SI) to achieve the desired volume fraction *f*. This permits a large range of volume fractions without significant overlaps between fibers.

In order to lay out discrete fibers throughout the domain, we seed the domain with fibers of specified *α* and *f* aligned at a mean orientation identified by angles *θ*_*i*_^*m*^ and *θ*_*o*_^*m*^ obtained by averaging *θ*_*i*_(***x***) and *θ*_*o*_(***x***) respectively over the entire domain. Each fiber is then independently rotated about its centroid in order to match the optimal orientation at the centroid. This trivially preserves the fiber volume fraction at the macroscale, while there might be small variations at the microscale (see Fig. S7, SI). This approach works well if the gradients in fiber orientation vary gradually through the structure, but if there are regions with rotation angles deviating considerably from the packing direction, it can lead to excessive overlaps of rotated fibers. In such cases, we divide the packing domain into distinct regions and in each region, the fibers are independently packed using the above mentioned algorithm and the number of subdivisions is chosen to minimize the overlaps (see Fig. S6, SI for a specific example). We then check the projected fiber layout obtained by this algorithm (see Section 3.2, SI) for overlaps between the fibers and verify the fiber volume fraction. Figure 1e shows the final fiber arrangement in a sub-region of the design shown in Fig. 1d.

Once we have created a geometric realization of the microstructure through the entire structure, we transform this into a representation that can drive a 3D printer to fabricate it. We used a Stratasys Connex3 Objet500 printer which uses material jetting to deposit drops of multiple materials (here we use two – stiff (Vero) and soft (TangoPlus)) across a layer in a rectangular array with individual pixels of 42.3 × 84.7 μm. Immediately after depositing drops, a wiper sweeps over them to smooth them into a continuous film with a uniform thickness of 30 μm, and subsequently the layer is photocured with ultraviolet light. In this way the printer builds up a 3D solid, layer by layer with the result that the solid consists of voxels of dimensions 42.3 × 84.7 × 30 μm. The material jetting pattern executed by the 3D printer is described by a series of standard 2D bitmap files (.bmp); each layer is characterized by two bitmap files, one that describes the distribution of soft material and the other that describes the distribution of stiff material. This way, each pixel in a layer can be populated with either stiff, soft, or no material. We use Monolith, a voxel based modeling tool^42^ to translate our geometric microstructure representation into the distribution of stiff and soft material on a discretized 3D grid spanning a watertight mesh surface of the exterior of the optimal structure (obtained from the design automation process – see Section 3.3, SI). It does this by slicing the 3D solid into slices (of 30 μm thickness in our work) and then carrying out a rasterization process familiar in 2D image processing to convert each slice into a high-resolution bitmap. Figure 1f shows a zoomed-in part of a layer of the voxelized representation of the sub-region shown in Fig. 1e; the effects of voxelization on the cylindrical shape are clear. In order to obtain a voxelized representation that is a reasonable mechanical equivalent of the actual fiber geometry, as determined by our mechanical tests of composite samples, we chose 2*r*_*f*_ = 0.36 mm which guarantees at least four voxels along the diameter in any orientation. A printed structure with a soft (TangoPlus) matrix and glassy polymer (Vero) fibers is shown in Fig. 1g. We also printed a structure for visualization with transparent and black colored materials for matrix and fiber respectively that is shown in Fig. 1f. For simplicity, the example in Fig. 1 is a 2D structure with a 3D microstructure. In this case no support material is required in the printing process. In general for a 3D structure it is necessary to also print a photopolymerizable gel-like support material in regions with overhangs or internal geometric features and in this case a final, analog, process of support removal by mechanical agitation or chemical dissolution is necessary.

**Figure 2:**
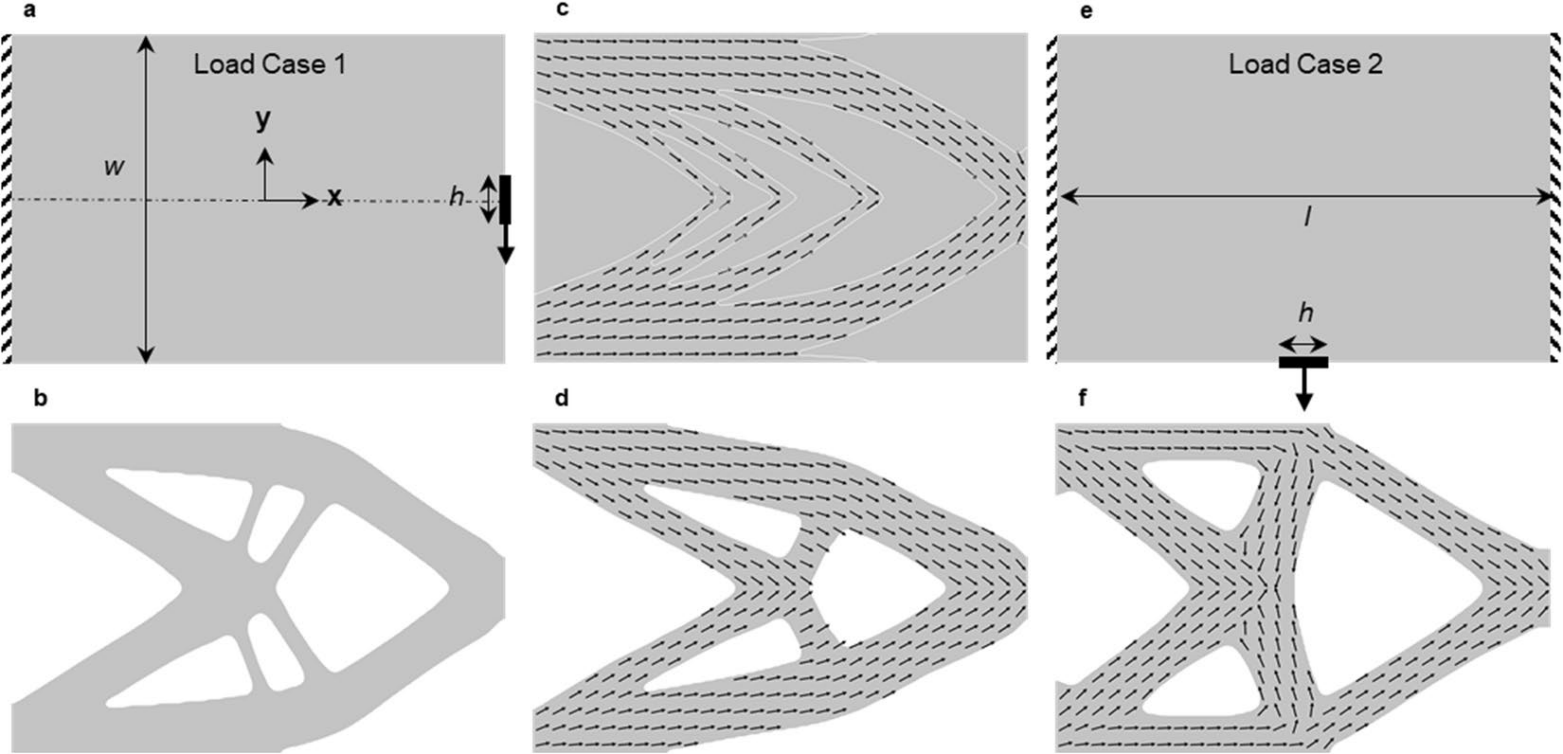
Structures with planar geometry (**a**) Design domain (*l* = 300 mm and *w* = 200 mm, *h* = 30 mm) and setup for a 2D cantilever beam (dashed line indicates design symmetry plane). (**b**) Case 1: macroscale topology optimization with a fixed microstructure which is an isotropic solid (**c**) Case 2: microstructure optimization with a fixed macroscale topology leading to optimal fiber distribution within a continuous matrix, and (**d**) Case 3: simultaneous macroscale topology and microstructure optimization. (**e**) Problem setup for a 2D double clamped beam. (**f**) Case 4: Same as (**d**) but optimized simultaneously for two different loading scenarios – a cantilever beam (**a**) and doubly clamped beam (**e**). In all the examples, *f* = 0.1 and *α = *10, and arrows indicate fiber orientations.

**Figure 3:**
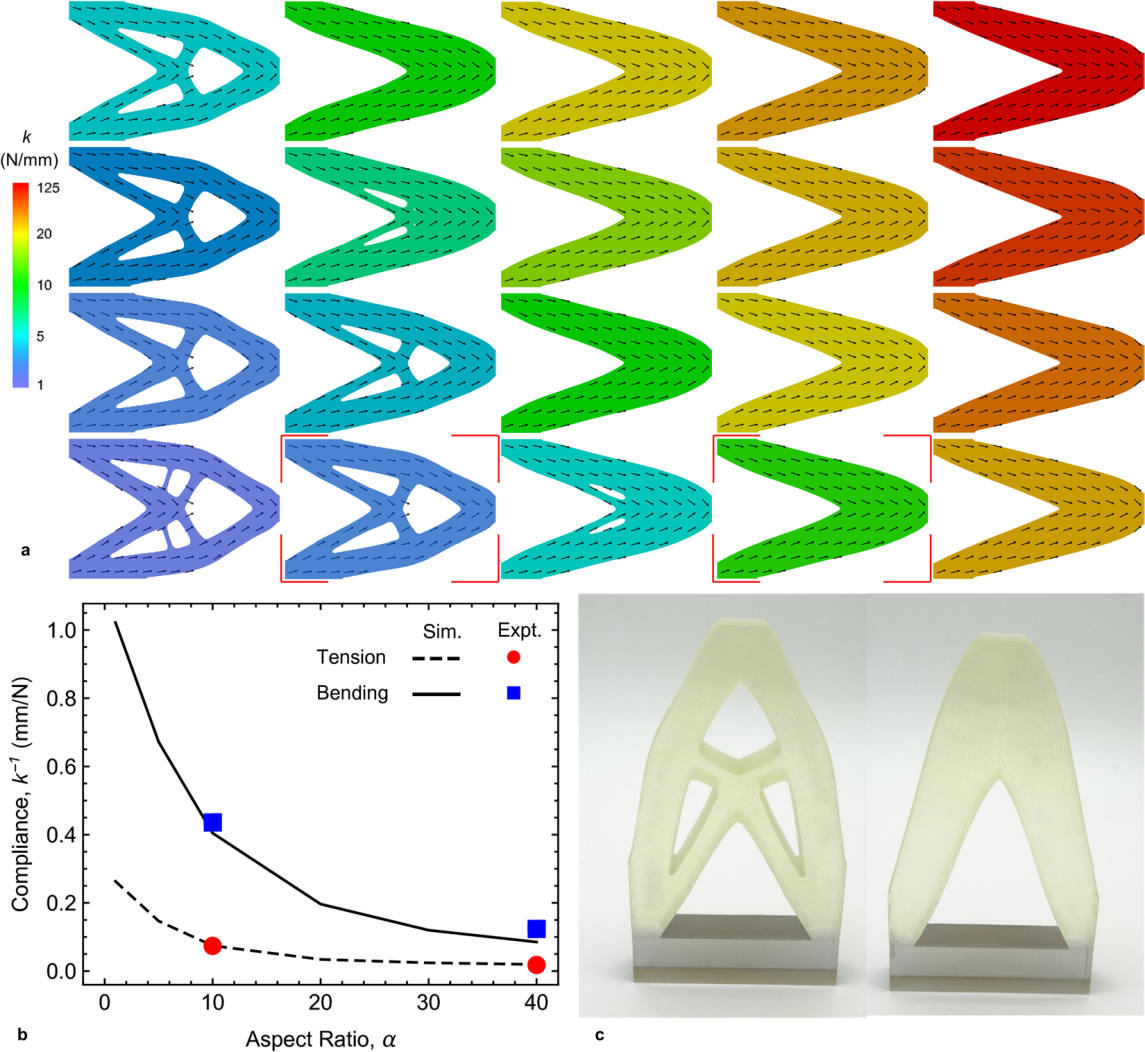
(**a**) Optimal designs of planar structures with different aspect ratios, *α* and volume fractions, *f* (*α* = 5, 10, 20, 40 and $\infty$ (continuous fibers) from left to right; *f* = 0.1, 0.2, 0.3 and 0.4 from bottom to top). The colors indicate the stiffness values (color legend on the left) and arrows indicate optimal fiber orientations. (**b**) Effect of aspect ratio on the compliance of the structures with a fixed *f* = 0.1 in both bending (solid curve, blue squares) and tension (dashed curve, red circles). (**c**) Printed structures of height 120 mm (excluding the transparent base) of the highlighted designs in (**a**) with *α* = 10 case on the left and *α* = 40 on the right.

### Optimal Design and Manufacture of Planar Composite Components

**Figure 4:**
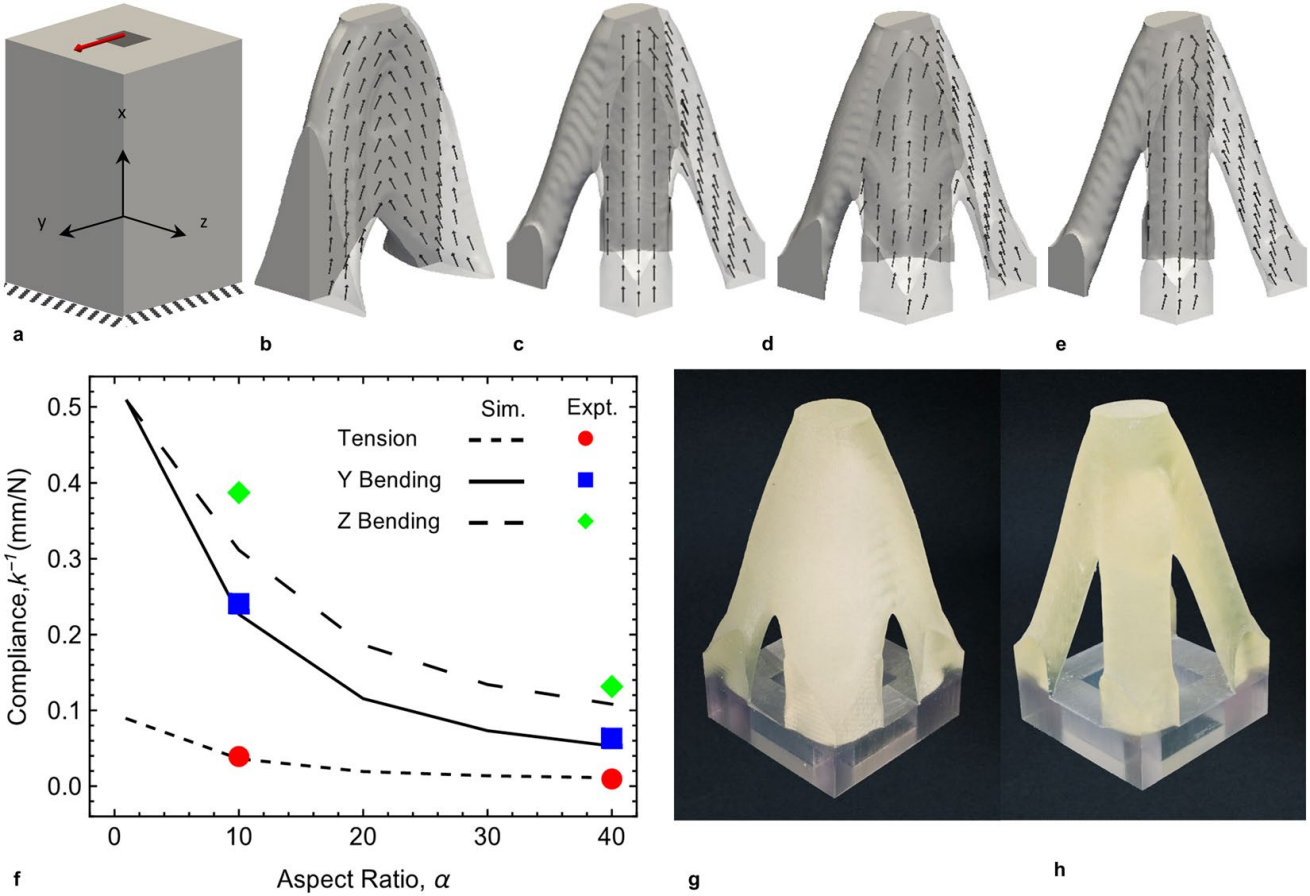
(**a**) Design problem setup of a 3D structure with a load along y-axis and the bottom surface clamped. Optimal designs obtained for (**b**) Case 1: 2-fold macro and micro symmetry imposed about xy- and xz-planes (**c**) Case 2: 4-fold macro and micro symmetry imposed about xy-, xz- and the plane bisecting xy- and xz-planes (**d**) Case 3: mixed macro (4-fold) and micro (2-fold) symmetry. Results in (**b**–**d**) are with *α* = 10 and *f* = 0.1. (**e**) Same as Case 3 (**d**) but with *α* = 40. In (**b**–**e**) the arrows indicate the optimal fiber orientations. (**f**) Effect of *α* on the compliance of the structures designed with the formulation of Case 3 in bending in the y direction (solid curve, blue squares), z direction (long dashed curve, green rotated squares), and tension (short dashed curve, red circles); curves are from simulations and symbols from experiments. (**g** and **h**) are printed structures for the results in (**d** and **e**) respectively; the height of the structures is 120 mm excluding the transparent base.

Figures 2–4 show applications of our approach to a series of examples. Figure 2 shows four planar geometry cases, each with a rectangular design domain, Ω with volume *V*_*Ω*_ and with traction loads applied over a small area (Fig. 2a,e). The design objective is to maximize the stiffness (minimize the strain energy, *U*) of the structure while constraining the volume of material used, V^*^ (V^*^ < *V*_*Ω*_). We assume plane stress and take the fiber distribution to be in the plane of the structure so *θ*_*o*_ = 0, and for simplicity we fix the fiber aspect ratio at *α* = 10 in all the scenarios leaving *ρ*, *f* and *θ*_*i*_ as possible design variables. From this setup, we formulate four design cases: in Case 1 we optimize the macroscale topology while keeping the microstructure fixed as an isotropic solid, while in Case 2 we fix the topology of the design domain, and optimize the spatial layout of the microstructure. Cases 3 and 4 optimize both the macroscopic topology and the microstructure for single and multiple load cases respectively. The formal design optimization problem can be stated as:

2 $$\begin{array}{cc}\mathop{min}\limits_{s} & U({\boldsymbol{u}}({\boldsymbol{s}}),\,{\boldsymbol{s}})\\ {\rm{s}}{\rm{u}}{\rm{b}}{\rm{j}}{\rm{e}}{\rm{c}}{\rm{t}}\,{\rm{t}}{\rm{o}} & V({\boldsymbol{s}})\le {V}^{\ast }\\  & {{\boldsymbol{s}}}_{l}\le {\boldsymbol{s}}\le {{\boldsymbol{s}}}_{u}\end{array}$$

We size the rectangular design domain with *l* = 300 mm and *w* = 200 mm (Fig. 2a) to achieve a separation of scales between the macroscopic structure and the microstructure, based on our finding that printed fibers of length *l*_*f*_ = 3.6 mm performed well. Later we relaxed this and designed and printed smaller macroscale samples and still achieved reasonable results in terms of their mechanical behavior. In the examples in Fig. 2, we constrain the volume of material to be V^*^ = 0.5 *V*_*Ω*._ In order to avoid noise and encourage a smooth variation of the design variables over the design domain, we regularized the optimization variables via a linear smoothing filter^43^. To obtain a near 0–1 design i.e., minimal intermediate densities, a projection filter^43,44^ (Section 2.2, SI) is applied on smoothed optimization variables used in defining the densities. In addition, the designs are constrained to be symmetric about x-axis, i.e., *ρ(x, y)* = *ρ(x*, −*y)* and *θ*_*i*_*(x, y)* = −*θ*_*i*_*(x*, −*y)*. The design, objective, and constraint histories during the optimization process for each scenario are presented in the SI (Section 4.1) to show the evolution and convergence of the designs.

Figure 2b shows the result for Case 1where the microstructure was fixed as an isotropic material (*f*(***x***) = 0, *θ*_*i*_ (***x***) = 0°) and the macroscale topology was optimized. This baseline result recovers the well-known classical topology optimization result which now exist in commercial design software and represents the state of the art in design for additive manufacturing. The complexity of even this simple result illustrates the power of the connection between topology optimization and additive manufacturing, as traditional manufacturing processes are ill-suited to fabricate it efficiently. Figure 2c shows the result for Case 2 where the volume of the design domain is fixed (*ρ*(***x***) = 1) but a fibrous microstructure (*f* and *θ*_*i*_) is designed to maximize the stiffness where 0 ≤ *f* ≤ 0.1 This example is representative of a large class of natural materials like animal tissue and plants where internal microstructure facilitates optimal performance and is often fibrous, spatially-variable, and anisotropic.

Figure 2d shows Case 3 where we simultaneously optimized the macroscale topology and the composite microstructure. For simplicity we fixed *f* = 0.1, so *ρ* and *θ*_*i*_ are the only design variables but we could let *f* vary as well to create more design freedom. Given the simplicity of the problem it is not surprising that the result shares similar features with Fig. 2b, but there are differences in the macroscopic topology and it is notable that the optimal solution aligns the stiff fibers along the structural members supporting the bending loads. Finally, Fig. 2e,f show the problem setup and solution for Case 4 where we design a structure to simultaneously maximize stiffness under two different loadings and boundary conditions; this could be extended to multiple load scenarios in a straightforward manner. We formulate the objective as a linear combination of the strain energies obtained by solving two different finite element problems – the cantilever in Fig. 2a and the doubly-clamped cantilever in Fig. 2e. Compared to Case 3 (Fig. 2d) the optimized design (Fig. 2f) places vertically-oriented fibers in the mid-section to make the structure sufficiently stiff locally under both load cases.

Figure 3a shows optimal structures (topology and fiber orientation) for the optimization setup in Case 3 (Fig. 2a,d) over a range of design problems where we fixed the fiber aspect ratio (*α* = 5, 10, 20, 40, ∞ (continuous fibers)) and volume fraction (*f* = 0.1, 0.2, 0.3, and 0.4). The color of each design depicts the structure stiffness (*k* = applied force divided by load-point displacement). The structure stiffness increases as both *f* and *α* increase. The solution tends to that of an isotropic solid in the lower-left corner. As the composite stiffness increases (with increasing *α* and/or *f*    ), the anisotropy increases and the macroscale topology tends toward a two-bar frame without holes, with fibers aligned along the bars (upper-right corner).

Figure 3b shows results from exercising the complete digital design and manufacture workflow by 3D printing two samples highlighted in Fig. 3a. We assessed the resulting microstructure of the samples by randomly sampling the actual printed fiber volume fraction throughout the sample and found that the spatial variation of the actual realized microstructure varied little from the optimal design (SI, Fig. S7). For a uniform composite with this fibrous microstructure, the aspect ratio variation from *α* = 10–40 results in about a 10x difference in macroscopic modulus (Fig. 1a; SI Table S5). We carried out mechanical tests of the behavior of each sample in bending (the loading the samples were designed for) and tension (which they were not designed to optimize) and in Fig. 3c we compare these to predictions of structure compliance (=*k*^*−1*^) obtained from simulations (also see Table S1, SI). Figure 3c includes results with different macroscopic sizes (300 × 200 mm, 210 × 120 mm and 120 × 80 mm, with fiber diameter = 0.36 mm) for the *α* = 10 case to challenge the strict requirement of scale separation (*l*_*f*_ ≪ *l*). The larger samples reasonably satisfy the scale separation requirement, but even though the smaller ones do not, we obtain reasonable agreement (<11% variation) between predicted and measured macroscopic bending and tensile structure stiffness of the (Table S2, SI).

### Optimal Design and Manufacture of 3D Composite Components

We extended the 2D optimization problem to a series of analogous 3D problems where we no longer fix *θ*_*o*_, thus allowing non-planar fiber orientations. Encouraged by the good results with the smaller 2D structures, and in order to minimize printing costs, we specified a design domain of 120 × 80 × 80 mm and a traction load is applied over a region of 19.6 × 19.6 mm in the *y*-direction (Fig. 4a). Thus the structure tends to bend about the *z*-axis. We restrict the designs such that *V* ≤ V^*^ = 0.25 *V*_*Ω*_. Unless otherwise mentioned, the rest of the optimization problem setup is same as that of the 2D problem.

With this setup, and while fixing *f* = 0.1 and *α* = 10, we formulated and solved three different design problems (Cases 1, 2, 3), the difference being the way design symmetries are specified in each to demonstrate how we can independently control the macroscale and microstructure design. Specifically, to control the macroscale design, i.e., the topology, we impose symmetry in the optimal solution for the material density *ρ*, independent of the fiber orientation. To control the microstructure design, we impose symmetry on the fiber orientation (*θ*_*i*_, *θ*_*o*_) independent of density. Details regarding the problem setup, symmetry boundary conditions, and results are provided in Section 4.2, Table S3, SI. For Case 1, we impose two-fold symmetry on both the macroscale and microstructure design. This is intended to create a structure with maximum stiffness as loaded, but if the structure were loaded with a force at the same location but in the *z*-direction, the stiffness would be expected to differ. The optimal result is shown in Fig. 4b and it is considerably stiffer when the force is applied in the *y*-direction (*k*_*y*_ = 5.43 N/mm) than in the *z*-direction (*k*_*z*_ = 0.72 N/mm). For Case 2 we impose 4-fold symmetry on both the macro and microscales with the goal of creating a structure that has the same mechanical response when loaded by forces in the *y*- or *z*-directions; the optimal solution is shown in Fig. 4c and the stiffness is *k*_*y*_ = *k*_*z*_ = 3.93 N/mm which falls between *k*_*y*_ and *k*_*z*_ of Case 1. We argue that these two cases reflect the widely adopted design principle that *form follows function* often attributed to the Roman architect and engineer Vitruvius. This is because the macroscopic topology (form) and microstructure are simultaneously optimally determined to lead to the desired stiffness (function). In Case 3 we show a simple example of how this principle can be challenged by imposing 4-fold symmetry on the macroscopic topology, but allowing more freedom in the microstructure by only imposing 2-fold symmetry. The optimal solution is shown in Fig. 4d. While macroscopically the structure appears like it would be equally stiff in response to loading in the *y*- and *z*-directions, the optimal microstructure gives rise to significant differences in stiffness with *k*_*y*_ = 4.41 and *k*_*z*_ = 3.21 N/mm. Here the function does not follow the form, at least the macroscopic form that is the basis of the design principle. The result of imposing the macroscopic symmetry, though, reduces the obtainable design stiffness *k*_*y*_ from Case 1, but it exceeds that of Case 2. These examples, while simple, illustrate the powerful design freedom that results when the design of the macro and microscales are decoupled. In Fig. 4e we show a result for the same problem formulation of Case 3 (mixed symmetry of macro and microscales), but with *α* = 40. We explore the effect of microstructure on performance in more detail in Fig. 4f where we plot the resulting stiffness for the formulation of Case 3 as a function of *α*. We show results for the compliance with a force applied in both the *y*- and *z*-directions as well as tension (force applied in the *x*-direction). Note that the structures were only optimally designed for a force applied in the *y*-direction.

Again we exercised the complete digital design and manufacture workflow by designing physical fiber microstructures and 3D printing the two samples from the results in Fig. 4d,e and these are shown in Fig. 4g,h. We carried out mechanical tests of the behavior of each sample in bending in both the *y*- and *z*-directions and tension. We plot these results in Fig. 4f and compare them to simulations. As with the 2D results we obtain good agreement between predicted and measured macroscopic behavior and demonstrate the macroscopic anisotropy in mechanical behavior due to the microstructure, i.e, the demonstration of function not strictly following form.

## Conclusion

We presented a new approach that integrates engineering design, material design, and product fabrication in a completely digital workflow that allows unprecedented product performance optimization, as well as efficiencies through the product development lifecycle. It can be adapted to myriad design, analysis, and fabrication technologies to simultaneously design material microstructure and macroscale structural topology and create highly-optimized products with specified performance. We demonstrated and experimentally validated this first-of-its-kind technology for a series of mechanical structures designed with multiscale topology optimization and realized by voxel-based multimaterial jetting additive manufacturing. While they illustrate many powerful features of the new approach, continued development incorporating multiple physical phenomena will unleash new, potentially transformational, capabilities.

## Electronic supplementary material

Supplementary Information

## Data Availability

The datasets generated and analyzed during the current study not included in this article (and its supplementary information) are available from the corresponding author on reasonable request.
