# Supplementary material for: Simultaneous Digital Design and Additive Manufacture of Structures and Materials
Source: Sci Rep. 2018 Oct 22;8:15560. doi: 10.1038/s41598-018-33454-3 (PMC6197205; doi:10.1038/s41598-018-33454-3)
Supplement: Supplementary file 1 — Supplementary Information [file 41598_2018_33454_MOESM1_ESM.pdf]

Simultaneous Digital Design and Additive Manufacture of Structures and Materials

Supplementary Information

Narasimha Boddeti[1,2], Zhen Ding[1], Sawako Kaijima[1], Kurt Maute[2], Martin L. Dunn[1,3*]
[1]Digital Manufacturing and Design (DManD) Centre
Singapore University of Technology and Design
[2]Department of Aerospace Engineering Sciences, University of Colorado, Boulder
[3]College of Engineering and Applied Sciences, University of Colorado, Denver

1. Digital Workflow Concept

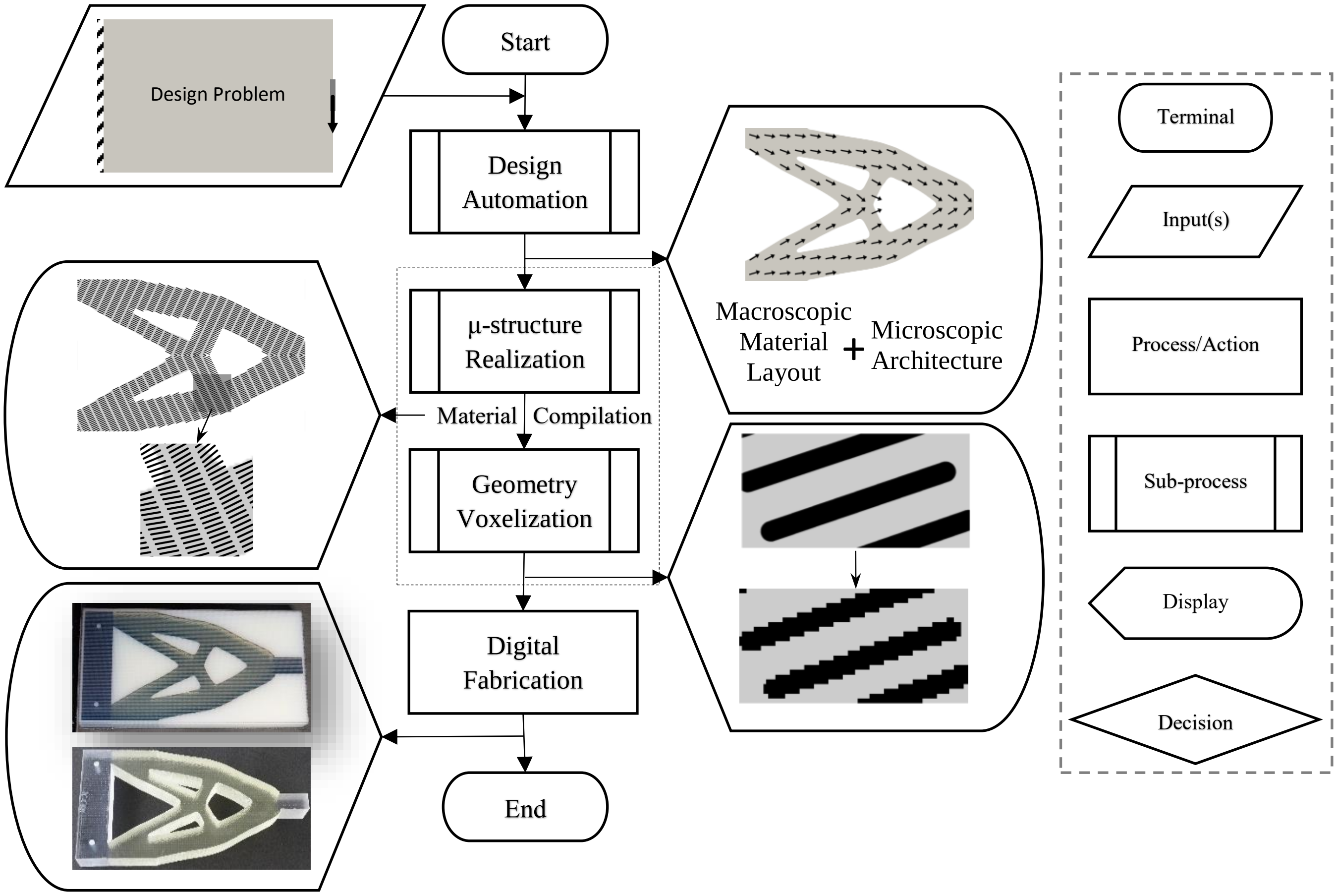

Fig. S1 Flowchart for simultaneous digital design and manufacturing of materials and structures.

We present, in Fig. S1, an alternative depiction of our digital design and manufacturing workflow shown in Fig. 1 of the main text with the help of a flow chart. For this and the subsequent flow charts, the symbols used and their meanings are shown in the box to the right of the flowchart. As described in the main text, the workflow begins with an input in the form of an engineering design problem that involves choosing the macroscale design domain, objectives, constraints and materials as well as the microstructure parametrization. The next step is the sub-process of *design automation*, the details and flowchart of which

are shown in Section 2 and Fig. S2 respectively. This process results in the macroscopic material layout and microstructural parameters at each macroscopic point. This is followed by *material compilation* process wherein we first determine the microscale geometry and distribution of the fibrous material via a *microstructure realization* algorithm (see Fig. S4, Fig. S6 and Section 3.1). This is followed by the *voxelization* sub-process (see Fig. S8 and Section 3.3) which then leads to *digital fabrication* via additive manufacture of the material and structure. The results of each process/sub-process are shown to either the left or right using the display element of the flowchart. In this text, we describe in full detail each process/sub-process involved in the workflow. Here and subsequently in this text, we use Case 3 of planar problems described in the main text (result in Fig. 2d) to illustrate workflows of various processes and sub-processes. The organization of the rest of this text follows the work flow shown in Fig. S1.

2. Design Automation

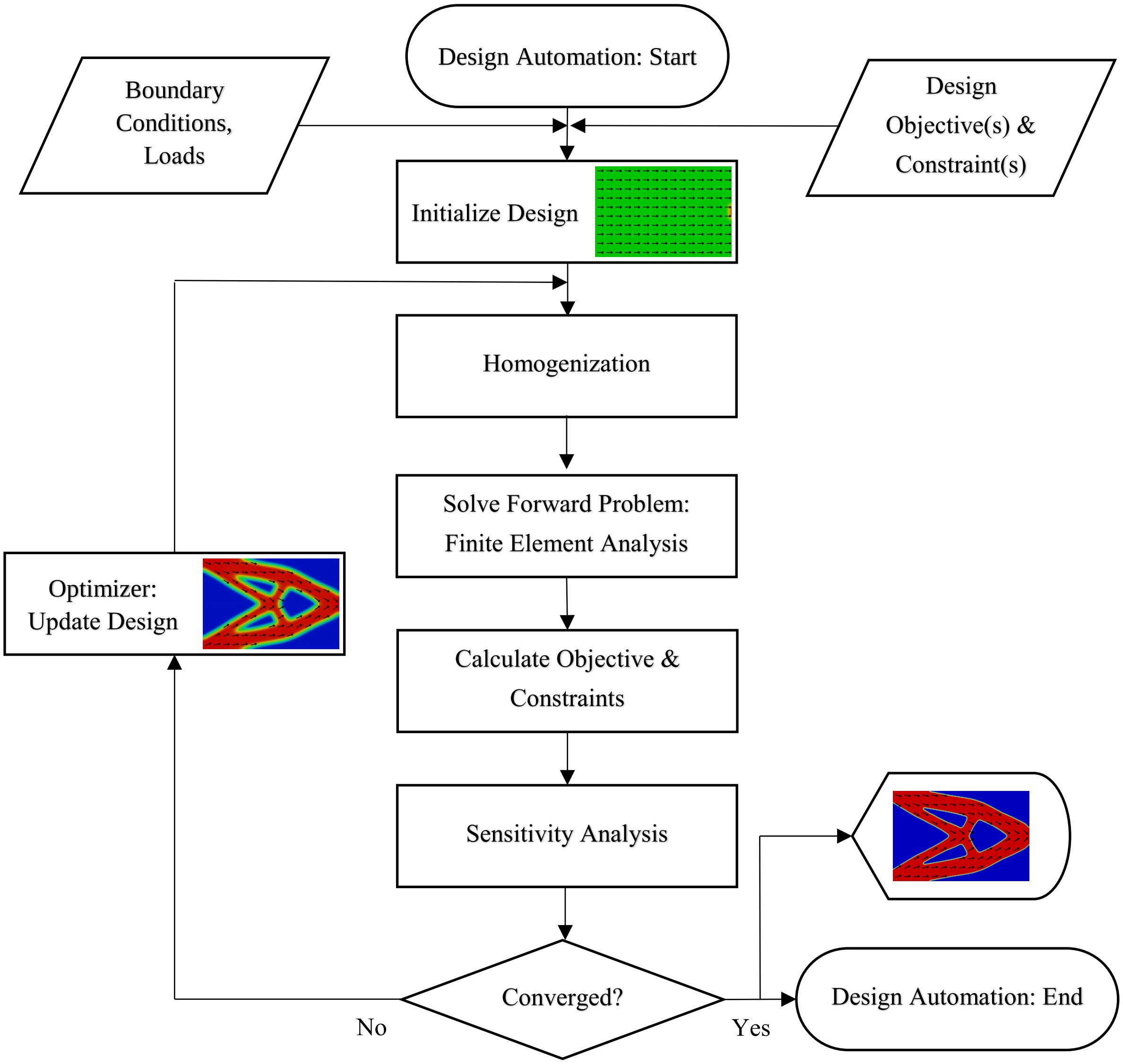

Fig. S2 Workflow for design automation via topology optimization.

In this section, we describe computational design automation via topology optimization and the intermediate steps involved within in detail (viz. *homogenization, forward analysis and sensitivity analysis)*. For the sake of clarity, we reiterate the general mathematical form of the topology optimization problem described in the main text. The goal here is to simultaneously find an optimal macroscale material layout i.e., topology and optimal microstructure:

$$\begin{aligned} \min_{\bar{s}} \quad & z(\bar{u}(\bar{s}), \bar{s}) \\ \text{subject to} \quad & g_i(\bar{u}(\bar{s}), \bar{s}) \leq 0 \qquad i = 1, \dots, m \\ & h_j(\bar{u}(\bar{s}), \bar{s}) = 0 \qquad j = 1, \dots, n \\ & \bar{s_l} \leq \bar{s} \leq \bar{s_u} \end{aligned} \tag{S1}$$

Here, as in the main text, z is the objective, g_i and h_j are the inequality and equality constraints and $\bar{s}$ the optimization variables vector with lower and upper bounds of $\bar{s_l}$ and $\bar{s_u}$. $\bar{u}$ is the displacement field obtained by solving the elastostatics equilibrium condition, $\nabla\sigma(\bar{u}) + \bar{b} = \bar{0}$ via finite element method (σ is the stress tensor and $\bar{b}$ is the body force vector). The stresses, σ are related to strains, ϵ and thus displacements through Hooke's law for continuous media, $\sigma = \mathbf{C}(\bar{s})\epsilon(\bar{u})$ ($\mathbf{C}$ is the material stiffness tensor). The material stiffness tensor, $\mathbf{C}$ depends on the local microstructure. Following density based SIMP (Solid Isotropic Material with Penalization) approach, $\mathbf{C}$ is interpolated between void and material using the normalized density, ρ. The distribution of ρ determines the topology with $\rho = 0$ implying void or non-existence of material and $\rho = 1$ that of existence. The objective, the constraints, and the displacement field are all functions of the design variables (ρ, and microstructural parameters) which in turn depend on the optimization variables.

Fig. S2 shows the flowchart for our design automation approach based on topology optimization. The design objective and constraints extracted from the engineering design problem along with mechanical loads and boundary conditions constitute the inputs to the workflow. The inputs also include the choice of the microstructure i.e. embedded short fibers in a matrix. The optimization problem is setup after identifying the appropriate design and optimization variables. After initializing the optimization variables and thus the design variables to meaningful values and extracting the material properties via a homogenization scheme, we perform *forward analysis* where we employ the finite element (FE) method to solve the discretized elastostatics equations to obtain the equilibrium displacement field. The objective and constraints are evaluated using the results obtained from FE analysis. This is followed by *sensitivity analysis* where we calculate design sensitivities i.e., objective and constraint gradients with respect to the optimization variables. A gradient based optimizer then utilizes the design sensitivities to update the optimization variables and hence the design variables. We extract the new material properties again from the updated design variables via the homogenization scheme and iterate the process until convergence. Discussion on the finer details of the homogenization scheme, forward problem setup, and sensitivity analysis follows.

2.1 Mori-Tanaka Homogenization Scheme

The Mori-Tanaka homogenization approach[1,2] provides analytical estimates for our choice of the microstructure, fiber-based composites. We adopted the Mori-Tanaka approach because: i) the theoretical underpinnings and limitations of the method are understood and reasonable for approximate solutions,[2,3] ii) it has been shown to deliver good predictions of the effective elasticity, other tensorial properties, when compared with experiments[3–6] and high-fidelity numerical simulations;[7] and iii) it is expressed in analytical, albeit complicated, form which is advantageous given the computational efficiency gains when compared to numerical homogenization techniques. For the special case of a composite consisting of two isotropic phases, aligned spheroidal reinforcement (fibers) in a matrix, the effective transversely isotropic stiffness tensor, $\mathbf{C_l}$ of the composite is given by:

$$\begin{aligned}\mathbf{C_l} &= \mathbf{C_l}(\alpha, f, \mathbf{C}_\mathrm{m}, \mathbf{C}_\mathrm{f}) \\ &= \mathbf{C_m} + f(\mathbf{C_f} - \mathbf{C_m})\mathbf{A^{MT}}\end{aligned} \tag{S2}$$

In eq. (S2), f is the reinforcement volume fraction, α is the reinforcement aspect ratio, $\mathbf{A^{MT}}$ is the Mori-Tanaka strain concentration tensor, $\mathbf{C}_\mathrm{m}$ and $\mathbf{C}_\mathrm{f}$ are the isotropic stiffness tensors of the matrix and fiber phases. The Mori-Tanaka strain concentration tensor is given by:

$$\mathbf{A^{MT}}(\alpha, f, \mathbf{C}_\mathrm{m}, \mathbf{C}_\mathrm{f}) = \mathbf{A^{dil}}\left((1-f)\mathbf{I} + f * \mathbf{A^{dil}}\right)^{-1} \tag{S3}$$

Here, $\mathbf{I}$ is the fourth order identity tensor such that $\mathbf{I}\,\mathbf{A} = \mathbf{A}\,\mathbf{I} = \mathbf{A}$ and $\mathbf{A^{dil}}$ is the dilute strain concentration tensor, which is in turn given by:

$$\mathbf{A^{dil}}(\alpha, f, \mathbf{C}_\mathrm{m}, \mathbf{C}_\mathrm{f}) = (\mathbf{I} + \mathbf{S}\,\mathbf{C_m^{-1}}(\mathbf{C_f} - \mathbf{C_m}))^{-1} \tag{S4}$$

Here, $\mathbf{S} = \mathbf{S}(\alpha, \nu_m)$ is the Eshelby tensor[8,9] which is a function of only the aspect ratio, α and the Poisson's ratio of the matrix material, ν_m. The stiffness tensor, $\mathbf{C_l}$ is defined in a local coordinate system attached to the fiber axis and the stiffness tensor in the global coordinate system, $\mathbf{C} = \mathbf{C}(\alpha, f, \theta_i, \theta_o, \mathbf{C}_\mathrm{m}, \mathbf{C}_\mathrm{f})$ is obtained via standard tensor rotation transformations of $\mathbf{C_l}$, where θ_i and θ_o are the angles between the *x*-axes of the local and global coordinate systems in the *xy*-plane and *xz*-plane respectively**.** In the optimization problem, we define the microstructure parameters, $f, \alpha,$ θ_i and θ_o in terms of the optimization variables, $\bar{s}$ (more details in the following sub-section).

2.2 Forward Analysis and Filtering

We solve the optimization problem by discretizing the problem domain into finite elements. We associate the optimization variables with the nodes of the discretized finite element *mesh*; the design variables $\rho, \alpha, f, \theta_i$ and θ_o and thus the material stiffness tensor $\mathbf{C}(\rho, \alpha, f, \theta_i, \theta_o)$ with the elements. The discretized form of the elastostatic equilibrium can be described compactly by the residual vector equation:

$$\bar{R}(\bar{u}(\bar{s}), \bar{s}) = \bar{0} \quad \text{(S5)}$$

The global finite element stiffness matrix, K is then defined as $\mathrm{K} = \frac{\partial \bar{R}}{\partial \bar{u}}$. For linear elasticity, the residual equation is simply: $\mathrm{K}\bar{u} - \bar{f} = \bar{0}$, with $\bar{f}$ being the global force vector. *Forward analysis* then involves solving the system of equations (linear system in our case) $\bar{R} = \bar{0}$ to obtain $\bar{u}$.

We remarked in the main text that the design variables are all defined as functions of the optimization variables. This serves to normalize the bounds as well as regularize the design problem in order to achieve mesh independent results and a good 0-1 design i.e., a clear demarcation between void and material. We employ standard regularization techniques based on filtering of the optimization variables.[10–12] To ensure smooth designs, we use a linear smoothing filter[10] for the optimization variables. The linear smoothing filter (denoted by L) smears out any sharp transitions and gives smoothly varying fields:

$$L(\bar{s}) = W_e \bar{s} \quad \text{(S6)}$$

Here, W_e is the linear filter weight matrix and is defined as:

$$\begin{aligned} W_{e_{ij}} &= \frac{w_{ij}}{\sum_j w_{ij}} \\ w_{ij} &= \max\left(r_s - r_{ij}, 0\right) \end{aligned} \quad \text{(S7)}$$

Here, r_{ij} is the distance between centroids of elements indexed i and j and r_s is the specified smoothing radius.

Additionally, we apply a projection filter[10,12] on top of the linear smoothing filter when calculating the densities. The projection filter, P maps values between given lower and upper bounds to either the lower or upper bound. This ensures a crisp design with a sharp boundary and no intermediate values which is highly desired for densities to obtain a well-defined topology. It is defined as:

$$P(L(\bar{s})) = \frac{\mathrm{Tanh}(\beta\eta) + \mathrm{Tanh}\left(\beta(L(\bar{s}) - \eta)\right)}{\mathrm{Tanh}(\beta\eta) + \mathrm{Tanh}\left(\beta(1 - \eta)\right)} \quad \text{(S8)}$$

Here, β and η are projection filter parameters. When $\beta \to 0$, the projection filter approaches a linear function ($P(x) \to x$) shown by the black dashed line in Fig. S3. The same figure shows the behavior of the projection filter at various values of β. At the limit of $\beta \to \infty$, the projection filter approaches the Heaviside step function with $\eta = 0$. η then serves to shift the threshold along the horizontal axes. It is to be noted that the projection filter with $\eta = 0$ provides for control of the minimum feature size while our choice $\eta = 0.5$ does not (refer to Guest et al[10]); it just gives us a 0-1 design. The relationship between *physical* design variables ($\rho, \alpha, f, \theta_i$ and θ_o) and optimization variables ($\bar{s}$) is:

$$\phi(\bar{s}) = \phi_{min} + (\phi_{max} - \phi_{min})F(\bar{s}) \quad \text{(S9)}$$

Here, ϕ_{max} and ϕ_{min} are the maximum and minimum values of the design variable ϕ respectively. F is either a single ($F = L(\bar{s})$) or composite filter consisting of the linear smoothing and projection filters ($F = P\big(L(\bar{s})\big)$).

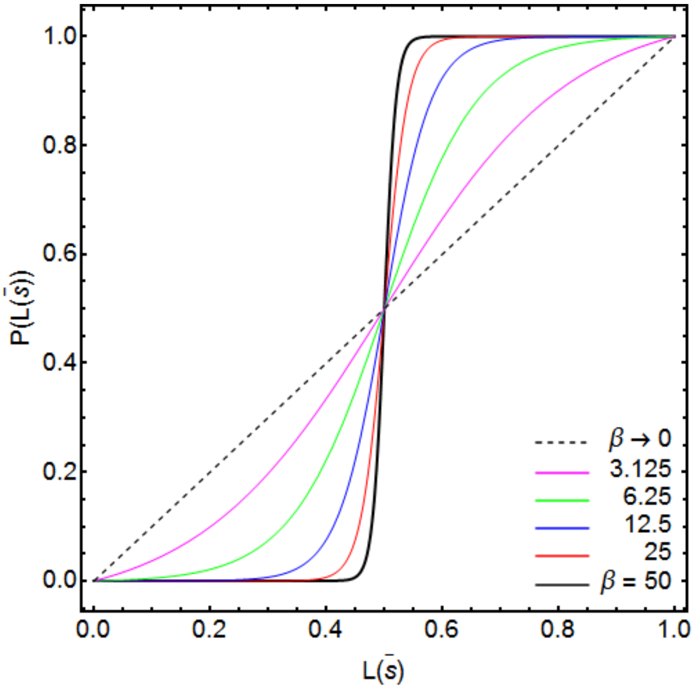

Fig. S3 Effect of β with $\eta = 0.5$ on the projection filter. The projection filter goes from being a linear function ($P(x) \to x$, $\beta \to 0$) to a shifted Heaviside step function as $\beta \to \infty$.

2.3 Sensitivity Analysis and Convergence

Sensitivity analysis involves calculation of the design *sensitivities* i.e., $dz/d\bar{s}$, $dg_i/d\bar{s}$ and $dh_j/d\bar{s}$. We obtain the sensitivities using the commonly used *adjoint* method. Note that with static equilibrium i.e. $\bar{R} = 0$:

$$\begin{aligned} \frac{d\bar{R}}{d\bar{s}} &= \frac{\partial \bar{R}}{\partial \bar{u}}\frac{\partial \bar{u}}{\partial \bar{s}} + \frac{\partial \bar{R}}{\partial \bar{s}} = 0 \\ \Rightarrow \qquad \frac{\partial \bar{u}}{\partial \bar{s}} &= -\mathrm{K}^{-1}\frac{\partial \bar{R}}{\partial \bar{s}} \end{aligned} \quad \text{(S10)}$$

Likewise, differentiating the objective, z and using eq. (S10):

$$\begin{aligned}\frac{dz}{d\bar{s}} &= \left(\frac{\partial z}{\partial \bar{u}}\right)^{\mathrm{T}} \frac{\partial \bar{u}}{\partial \bar{s}} + \frac{\partial z}{\partial \bar{s}} \\ &= -\left(\frac{\partial z}{\partial \bar{u}}\right)^{\mathrm{T}} \mathrm{K}^{-1} \frac{\partial \bar{R}}{\partial \bar{s}} + \frac{\partial z}{\partial \bar{s}} \\ &= -\bar{\lambda}^{T} \frac{\partial \bar{R}}{\partial \bar{s}} + \frac{\partial z}{\partial \bar{s}}\end{aligned} \tag{S11}$$

Here, $\bar{\lambda}$ is the solution of the linear system, $\mathrm{K}^{\mathrm{T}}\bar{\lambda} = \partial z/\partial \bar{u}$, which constitutes the adjoint problem. In the above equation, the partial derivatives, $\frac{\partial \bar{R}}{\partial \bar{s}}$ and $\frac{\partial z}{\partial \bar{s}}$ are calculated numerically by a finite difference scheme. The constraint sensitivities $\frac{dg_i}{d\bar{s}}$ and $\frac{dh_j}{d\bar{s}}$ are calculated similarly.

After calculating the sensitivities, we perform checks for optimality of the solution (in our case convergence of the objective and constraints such that the change is < 10^{-4} in the last 4 iterations). If the optimality checks do not succeed, a gradient based optimization algorithm (GCMMA[13], globally convergent method of moving asymptotes in our case) calculates a new set of optimization variables, $\bar{s}$. We then update the material properties to begin the next optimization iteration.

3. Material Compilation

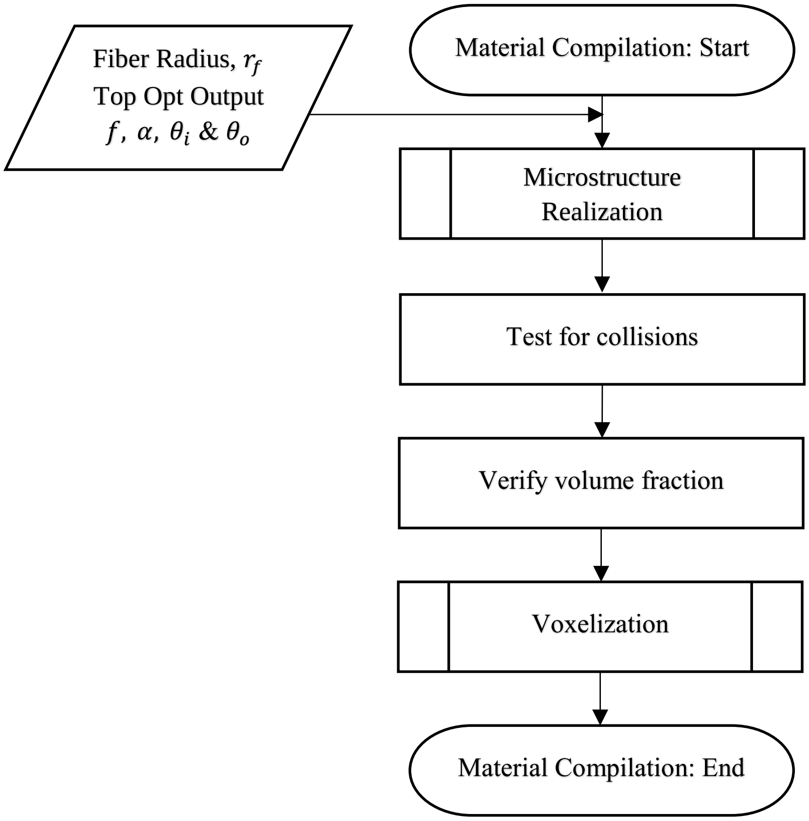

Fig. S4 Material compilation flow chart

The *material compilation* process involves two key components. The first is realization of the parametrized microstructure obtained from the design automation process i.e. exact geometry and distribution of the fibers within the matrix. We term this process as *microstructure realization*. The second is preparation of the realized geometry for fabrication via 3D printing accomplished by the *voxelization* process. The material compilation workflow is shown in Fig. S4. It starts with microstructure realization, followed by checks to look for possible overlaps or collisions amongst the generated fibers, verification of the volume fractions and ends with the voxelization process. Detailed discussion on each of these steps follows.

3.1 Microstructure Realization

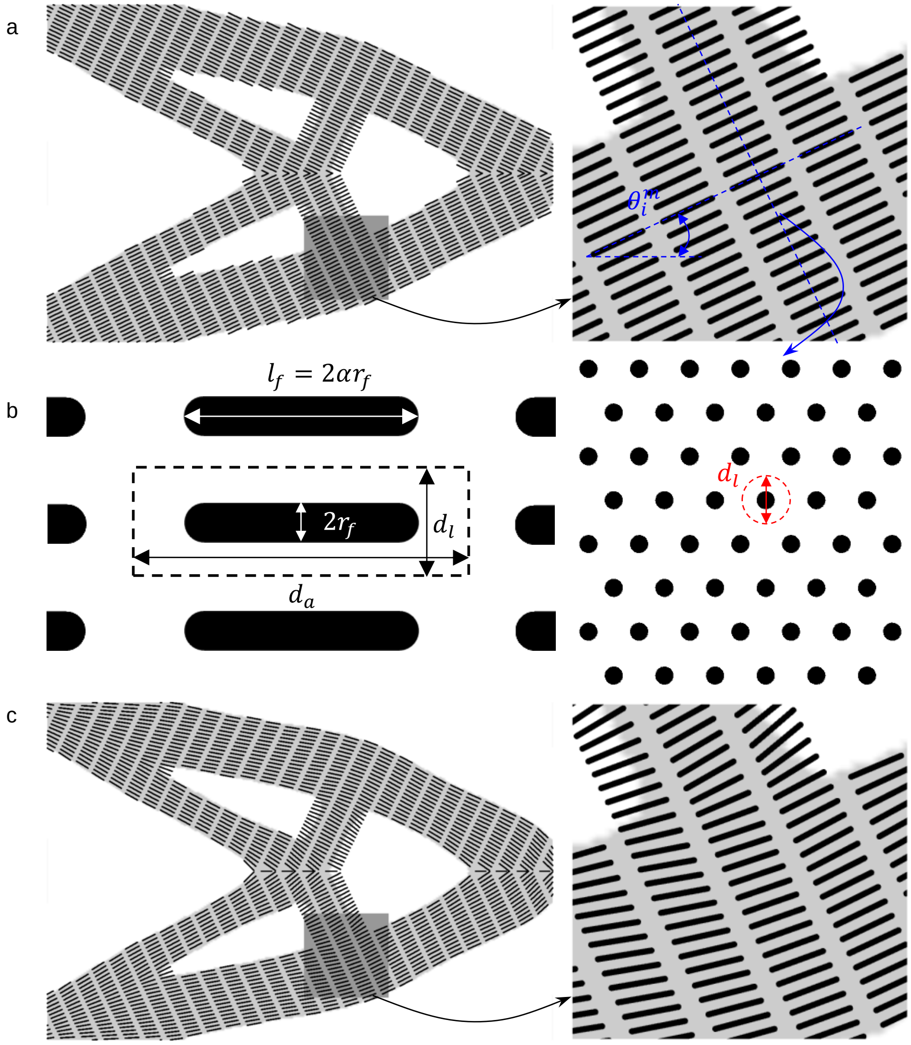

Fig. S5 (a) Initial arrangement of the fibers along the packing direction within the design domain on the left with a zoom-in on the right. (b) Schematics of the arrangement and various dimensions of the fibers in a plane parallel to the fiber axis (left) and hexagonal packing of the fibers in a plane perpendicular to the fiber axis. (c) Final arrangement of the fibers with their axes aligned along the optimal orientations.

We initiate the discussion of the microstructure realization algorithm with a simple example, case 3 of the planar structures as mentioned before, and then describe the process for a more general case. The objective, as previously stated, is to actualize the parametric microstructure distribution (α, f, θ_i and θ_o) obtained from the design automation step to an arrangement of short fibers that best approximates the mechanical behavior of the microstructure.

In the main text, we stated that the fibers are initially arranged in a hexagonal packing pattern and separated by distances $d_a > l_f$ and $d_l > 2r_f$: the axial and lateral distances respectively from the centroid of any given fiber and the centroids of immediately neighboring fibers (r_f = fiber radius, l_f = fiber length; see Fig. S5b). Alternatively, we can interpret that the fibers are encapsulated in an imaginary cylinder of diameter d_l and length d_a as shown in Fig. S5b. The goal then is to arrange these imaginary cylinders in an efficient manner such that a given volume fraction can be achieved. We choose hexagonal close packing as it is the densest for packing cylinders with the maximum possible packing density of $\pi/\sqrt{12}$. Noting that for the fiber and the imaginary cylinder containing it, the ratios of their volume fractions ($\frac{f}{\pi/\sqrt{12}}$) and the volumes ($\frac{V_{fib}}{V_{cyl}}$) should be equal, we arrive at eq. (S12) which relates the packing parameters, d_a and d_l with fiber radius, r_f and volume fraction, f.

$$\frac{V_{fib}}{V_{cyl}} = \frac{f}{\pi/\sqrt{12}} = \frac{2\left(\alpha - \frac{1}{3}\right) r_f^3}{d_l^2 d_a} \tag{S12}$$

We specify d_l and calculate d_a using eq. (S12). This leads us to a fiber arrangement as shown in Fig. S5a with the fibers initially arranged along the packing orientations, θ_i^m and θ_o^m (= 0 in this case). The packing orientations, as mentioned in the main text, are obtained by averaging $\theta_i(\boldsymbol{x})$ and $\theta_o(\boldsymbol{x})$ over the non-symmetric part(s) of the design (otherwise the average will trivially be zero). The same figure also shows the fibers in their final rotated configurations (Fig. S5c). We achieve the rotations by first obtaining the interpolated values of the orientation fields, $\theta_i(\boldsymbol{x})$ and $\theta_o(\boldsymbol{x})$ at the centroid of the fiber and then rotating the fiber about the centroid after having accounted for the initial rotation. We stagger the fibers from layer to layer for the 2D structures but not for 3D structures which otherwise leads to excessive collisions between fibers. Additionally, we introduce randomness in the fiber positions by rigidly translating them in a random direction by a small distance before rotating the fibers to their final configuration. These random perturbations and the staggering helps in not creating large gaps between the fibers that might create stress concentrations in the matrix material. However, for the example shown in Fig. S5, we did not introduce any randomness for the sake of clarity.

We now describe the more general workflow with the help of a more complicated example, case 4 of the optimal design with planar structures result of which is shown in Fig. 2f in the main text. The formal flowchart is shown in Fig. S6 along with results from the intermediate steps. We start with the abstract microstructure parameters obtained directly from the multi-scale design automation step. We divide the

optimized macroscale domain into multiple regions if the range of orientations present is large, for example 0 to 90°. This decision to divide and the number of divisions are both based on the number of resulting fiber overlaps/collisions which need to be minimized. For example, we divide the domain into two regions for the case shown in Fig. S6 such that the regions with orientation angles greater than 45° and less than 45° are separated. The divided regions are independently processed from here on. We calculate the packing directions for each region, fill them using hexagonal close packing and then rotate the fibers about their centroids to complete the microstructure realization process.

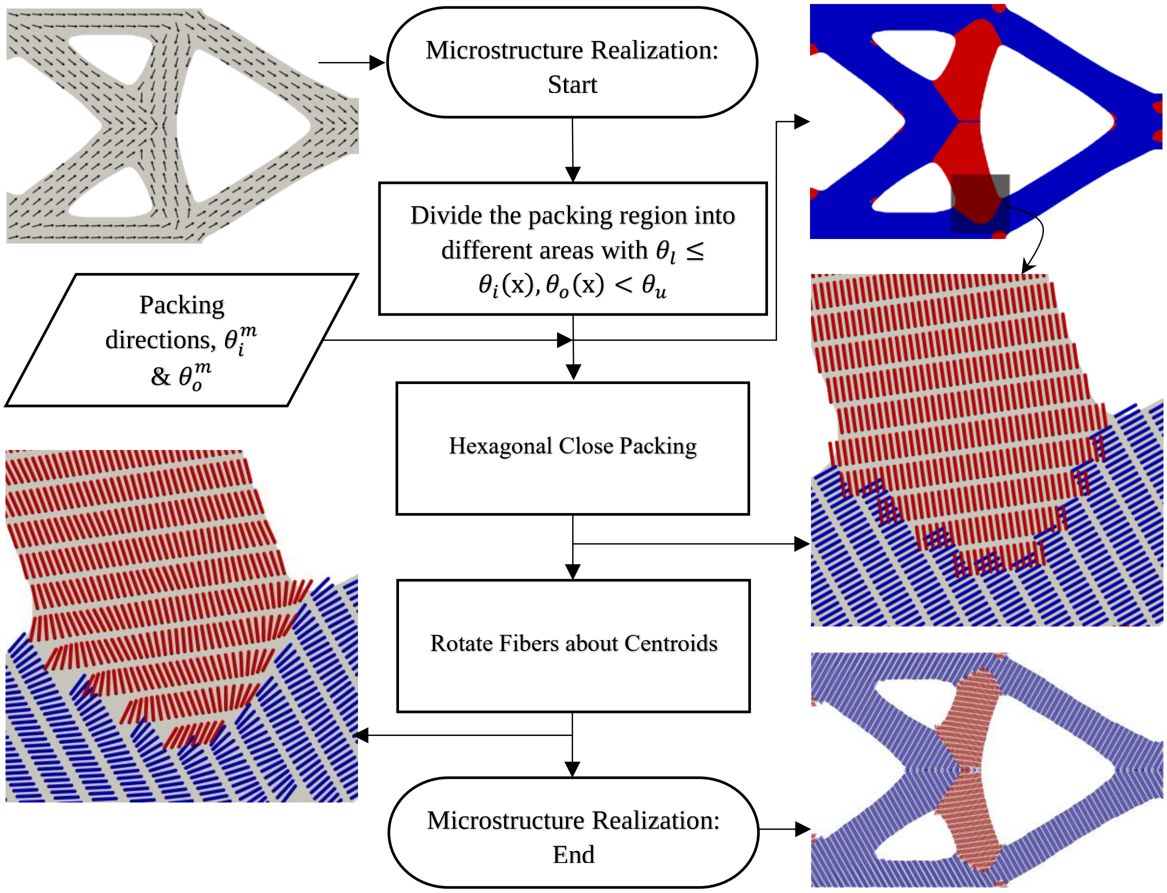

Fig. S6 Microstructure Realization flowchart with an example, red and blue coloring here indicate regions with $\theta_i > 45^o$ and $\theta_i \leq 45^o$.

3.2 Collision Tests and Volume Fraction Verification

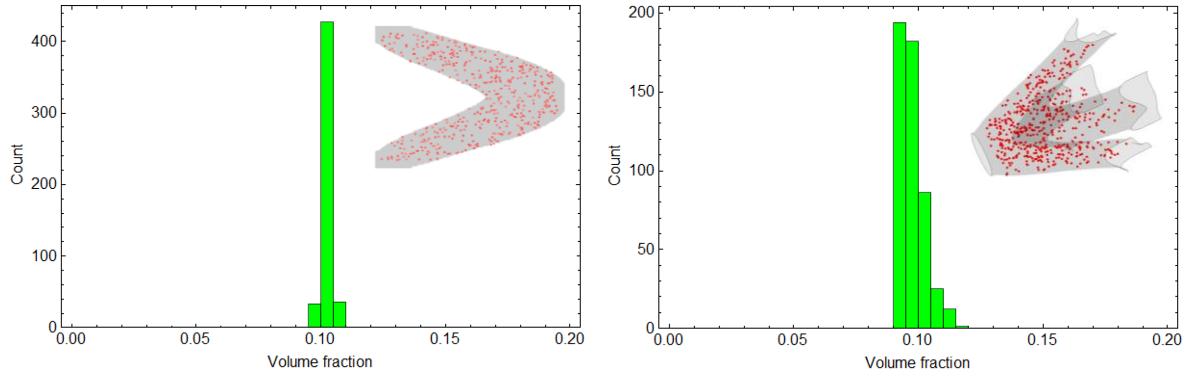

Fig. S7 Histogram of volume fractions of sampled volumes for $\alpha = 40$ *case in 2D (left) and 3D (right). Insets show the midpoints of the sampled cubical volumes of size 20 mm.*

We subject the fiber packing configuration to tests to check for collisions and verify the uniformity of the fiber volume fraction. For this purpose, we assumed that the end caps are flat surfaces instead of spherical caps, which would simplify the collision check algorithm but overestimate the number of collisions. The algorithm involves iterating through all the fibers while checking for possible overlap/collision between the current fiber and its nearest neighbors. The nearest neighbors are obtained by searching for fibers with start, end or midpoints within a specified search radius of the start, end or midpoint of the current fiber. The collision detection algorithm[14] identifies all possible scenarios of collisions based on the cylindrical geometry of the fibers. The algorithm sieves through most fiber pairs with a simple preliminary check. This preliminary check works by obtaining the minimum distance (d_{min}) between axes of the fibers in question. If $d_{min} \geq 2r_f$, there is no possibility of a collision, which accounts for most fiber pairs. If $d_{min} < 2r_f$, more nuanced checks are performed, the details of which can be found in Ketchel and Larochelle.[14]

Excessive collisions (> 5% incidence), despite division of the domain to minimize them as illustrated in Fig. S6, would indicate sharp gradients with high frequency in the optimal orientation fields ($\theta_i(\mathbf{x})$ & $\theta_o(\mathbf{x})$). This might be a result of inadequate regularization i.e. smoothing of the optimization variables, $\bar{s}$ associated with orientation design variables. If inadequate regularization is not the case, a potential solution is to smoothen the optimal orientation fields ($\theta_i(\mathbf{x})$ & $\theta_o(\mathbf{x})$) and repeat through previously mentioned steps in the microstructure realization flowchart (see Fig. S6). During this smoothing operation, the values of θ_i and θ_o are essentially mapped onto a suitably chosen coarser grid via averaging which then should smooth out the field by reducing the gradients. This smoothing solution, of course, comes at the cost of losing accuracy in the performance of the design which we can easily quantify via FE analysis to check if it is acceptable.

To verify the uniformity of fiber volume fractions, we randomly sample, within the optimal macrostructure, 500 cubical volumes whose side length is chosen based on the fiber size (e.g. 20 mm for $\alpha = 40$). For each cubical volume, we enumerate the total number of fibers that are fully or partially within a given cubical volume. For fibers completely within the cube, the volume is the sum of the volume of the two end caps and the cylinder in between ($= 2(\alpha - 1/3)\pi r_f^3$). For partial fibers, the volume is approximated as the sum of one end cap if one of the end points is in the cube and the fraction of the cylinder within the cube. The ratio of the total volume occupied by the fibers to the cube volume gives us the local fiber volume fraction. Fig. S7 shows histograms of calculated volume fractions for a 2D and a 3D structure with $\alpha = 40$. It can clearly be seen that we achieve a good uniform distribution of the fibers.

3.3 Voxelization

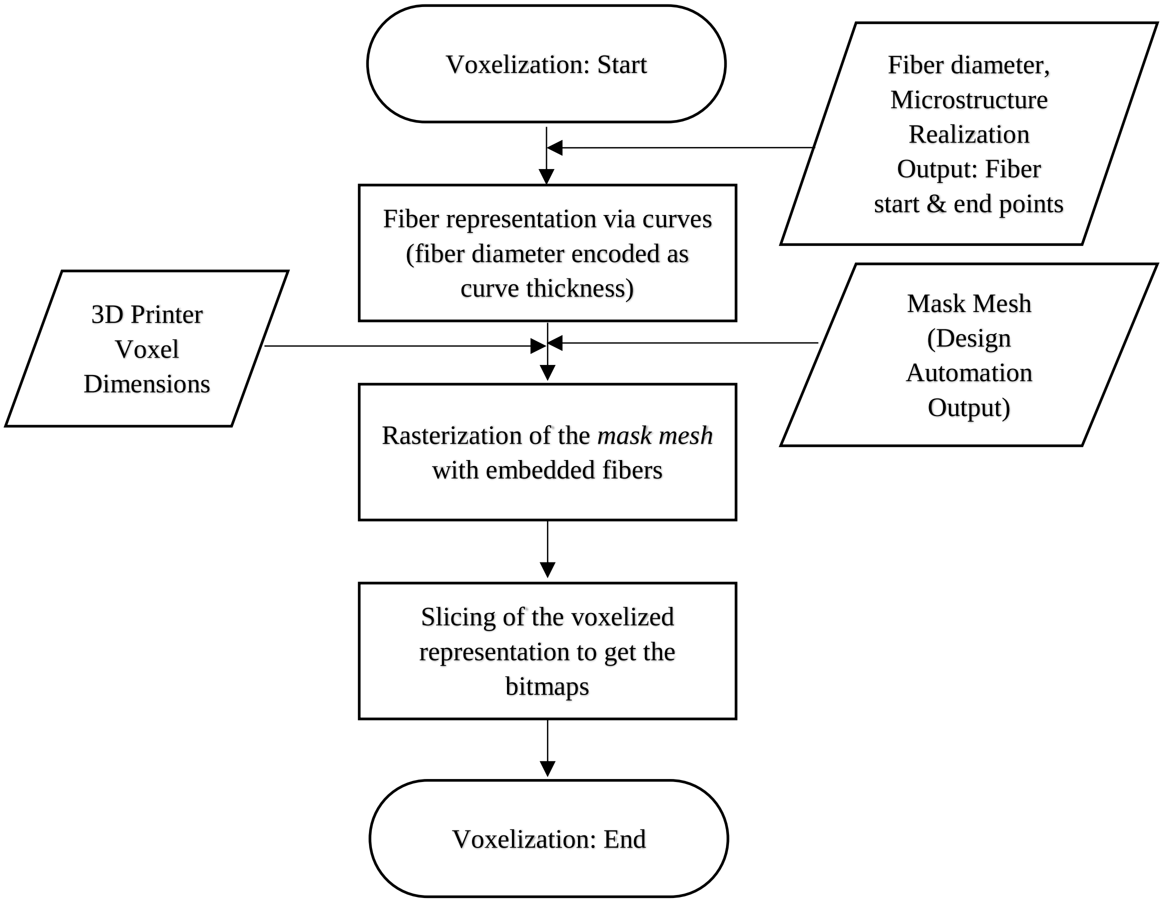

Fig. S8 Voxelization of geometry flowchart.

Depending on the 3D printing technology used, it is necessary to pre-process the geometry of the structure before printing. We developed a pre-processing algorithm for voxel-based 3D printing of fiber composites and adapted it for use with Stratasys Connex3 Objet500. The principle of operation of Objet500 is multimaterial jetting which is similar to inkjet printing. Instead of placing drops of ink on a paper, it involves precisely placing layers of individual droplets of different photopolymerizable resins onto a build

tray. A UV light source simultaneously cures each droplet layer as they are being placed. The printer specifically needs a set of bitmaps, one each for each material used, for each layer to be printed. The binary values (0 or 1) stored at each pixel in the bitmap associated with a material then indicates whether a droplet of that material is going to be jetted or not. The bitmaps when combined at the layer level and stacked gives the complete voxelized representation of the 3D structure to be printed.

We employed the voxel based modeling tool, *Monolith*[15,16] to obtain the voxelized representation of the composite structures obtained from the microstructure realization process. We then use the voxelized representation to generate the bitmaps necessary for voxel-based 3D printing. Fig. S8 shows the flowchart for the voxelization process. The process starts with compilation of inputs: fiber diameter, start and end points. The next step is to represent the fibers as line segments joining the start and end points and store the information in a CAD file (in Rhino's .3dm format, to be specific) needed by Monolith. Apart from this CAD file, Monolith requires the printer voxel dimensions (which are 42.33×84.66×30 µm) and a watertight mesh (called the *mask mesh*) of the macroscale topology obtained from the design automation step. Monolith first generates *distance fields* for the fibers within the mask mesh and ignores the fibers (or parts of the fibers) outside. The distance fields are gradient fields calculated based on the distance from the geometric primitive, a line segment in this case. An appropriate threshold value gives the boundary and hence the topology of the solid desired, cylinder in this case. Monolith then rasterizes the entire minimum bounding box containing the mask mesh, where rasterization refers to the process of converting the continuous representation of geometrical entities like lines, triangles, cylinders to discrete volume elements i.e., voxels. During this process, each voxel is also assigned a material based on its location. The voxels outside the mask mesh are regarded as void while the voxels inside are assigned either fiber or matrix material based on whether the voxel is within or outside the geometries generated by the distance fields. The mask mesh thus serves as a boundary and lets the rasterization algorithm ignore all entities outside this mesh. Monolith slices the rasterized entities to get the binary bitmaps needed to print with Objet500.

4. Simulations – Setup and Convergence

We present in this section, the details involved in the topology optimization problem setup including the finite element simulation setup. In addition, selected snapshots of the designs during optimization are presented to illustrate the convergence of designs towards the optimal structure. We divide this section into two sub-sections, one each for 2D and 3D problems respectively. In both the 2D and 3D problems, all the optimization variables (except the ones related to density) are bound to vary between 0 and 1. The lower limit of the density related optimization variables is set to 0.001 and not 0 to avoid numerical issues associated with zero stiffness finite elements. The orientation design variables, θ_i and θ_o can vary from 0° to 180°. We chose $\rho_{th} = 0.5$ for all the results as any material with $\rho < 0.5$ adds insignificantly to the overall stiffness of the structure as its material stiffness is only about 10% or less of the values of the bulk.

4.1 Optimal design of planar structures

For all the planar problems discussed in the main text, we used regular 4-noded plane stress linear quadrilateral finite elements of size, $h_e = 1.25$ mm to discretize the design domain (300×200 mm rectangle). We applied a linear smoothing filter with radius $8h_e$ on the optimization variables. A projection filter was applied on top of the linear filter after the first 200 steps. The value of β was doubled after every 200 steps from 3.125 for reasons explained later.

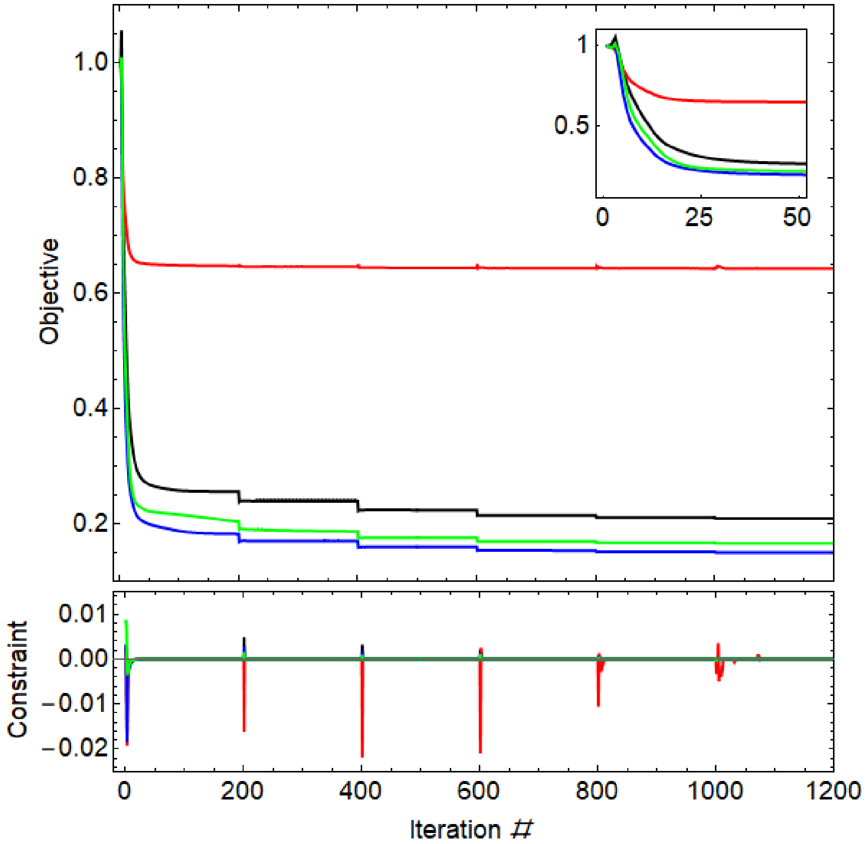

Fig. S9 Convergence history of the objective (top) and constraint (bottom) for the four cases from main text. Inset shows the first 50 iterations of the objective. Black curve – Case 1, Red – Case 2, Blue – Case 3, Green – Case 4.

We discussed four different cases in the main text: Case 1 is optimization with a fixed isotropic microstructure, Case 2 is optimization of microstructure with a fixed macrostructure, Cases 3 and 4 are multiscale optimization with one and two loading scenarios respectively. The objective and constraint convergence histories for these four cases are presented in Fig. S9. The designs at specific iterations are also shown in Fig. S10, Fig. S11, Fig. S12 and Fig. S13 for Cases 1, 2, 3 and 4 respectively along with arrows that depict the optimal fiber orientations and $\rho = 0.5$ iso-contour that demarcates void (isotropic matrix for Case 2) from the material.

We normalized the objective at the beginning of each simulation with the strain energy of the initial design (0th iteration). For Case 4 with two forward analyses, we still normalized the objective but made sure that the contribution from each forward analysis towards the objective is initially equal. Likewise, we chose the initial designs such that the constraint is satisfied and started with a value close to 0. The designs evolve to what looks like the final topology quickly as seen in Fig. S10-Fig. S13 but the designs have intermediate densities that are then gradually removed by the projection filter. As mentioned in Section 2.2, the purpose of the projection filter is to map the intermediate densities to either 0 or 1. This, if done in single step with a high β value, will lead to gross violation of the constraint, which might then lead the optimizer to a sub-optimal design or just slow convergence. To avoid this, we ramped the value of β gradually. As mentioned earlier, we did this after every 200 steps the effect of which can clearly be seen in the objective and constraint convergence histories.

Table S1 shows the stiffness values obtained from simulations and experiments (averaged) for structures optimized with setup in Case 3 and with $\alpha = 10$ and 40 in both bending, k_y and tension, k_x. The averaged stiffness values obtained from experiments for the three differently sized structures (300×200 mm, 210×120 mm and 120×80 mm) with $\alpha = 10$ are shown in Table S2.

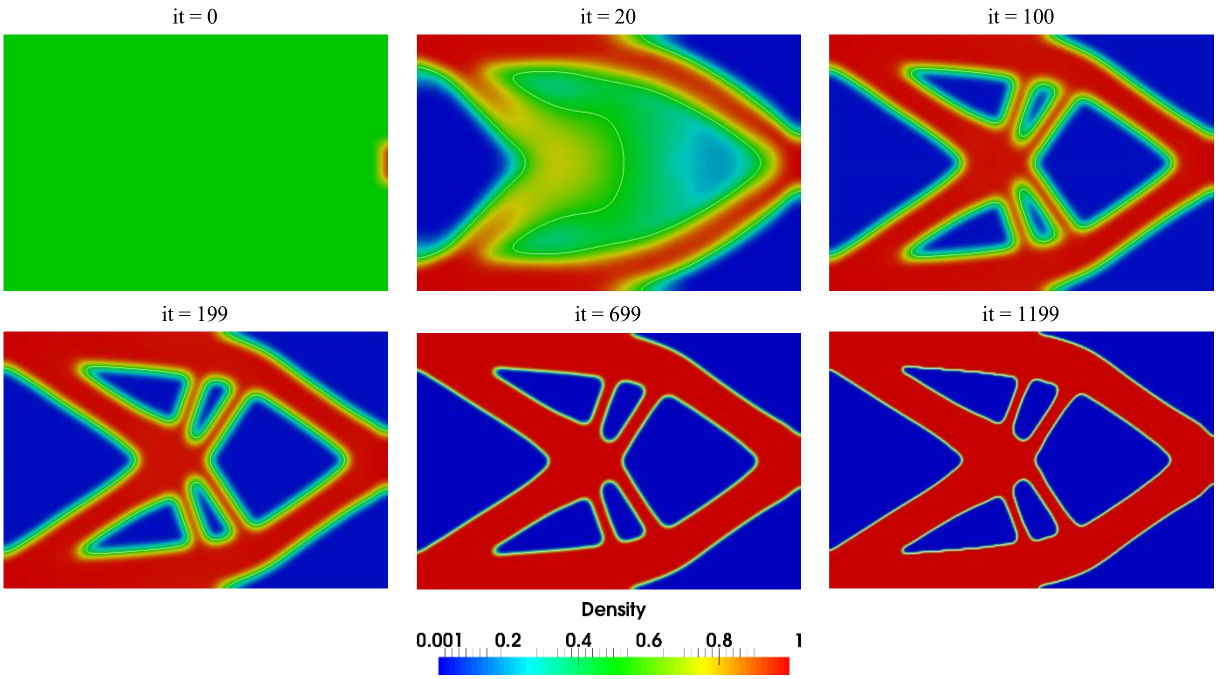

Fig. S10 Case 1: Designs at different iterations, including the first and last iterations. The white contour demarcates void (blue) from material (red).

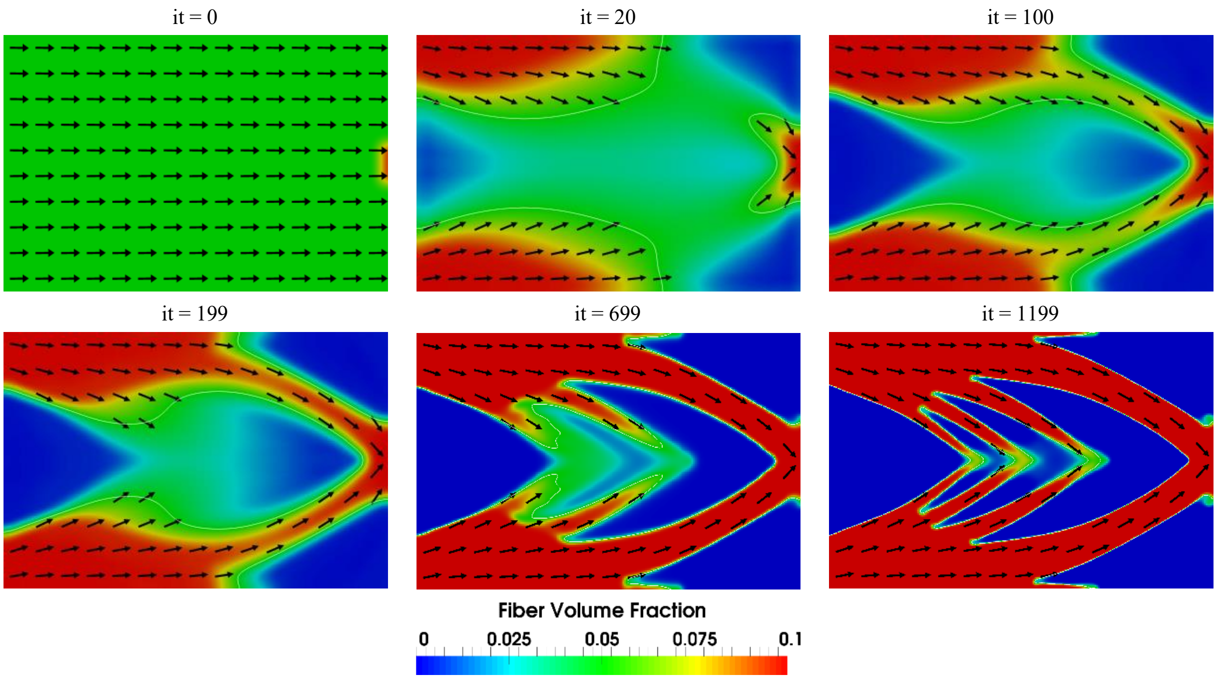

Fig. S11 Case 2: Designs at different iterations, including the first and last iterations. The white contour demarcates isotropic matrix material (blue) from the composite with $f = 0.1$ (red) and the arrows indicate optimal fiber orientations.

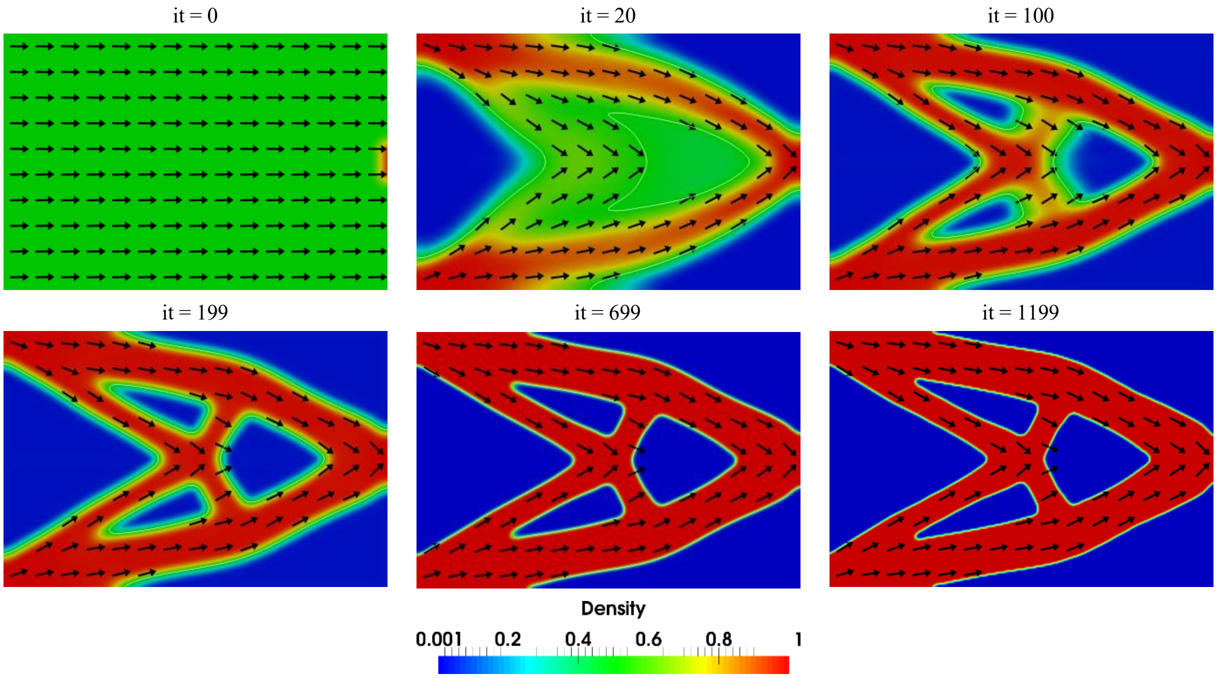

Fig. S12 Case 3: Designs at different iterations, including the first and last iterations. The white contour demarcates void (blue) from the material (red) and the arrows indicate optimal fiber orientations.

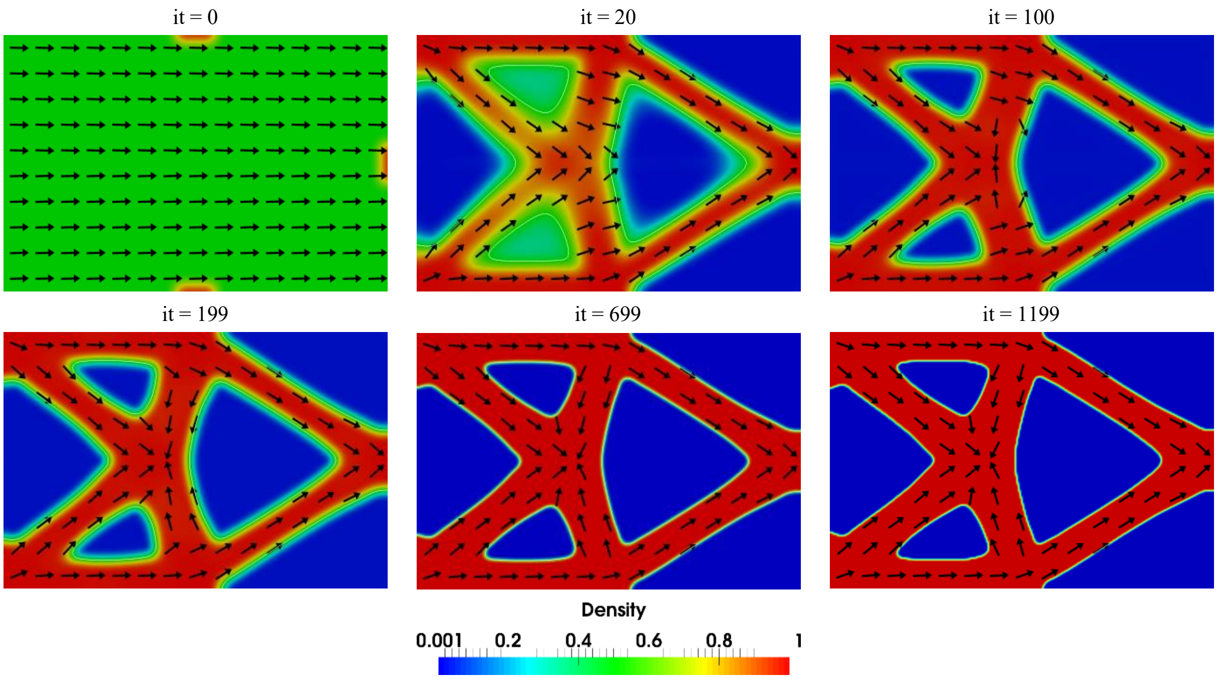

Fig. S13 Case 4: Designs at different iterations, including the first and last iterations. The white contour demarcates void (blue) from the material (red) and the arrows indicate optimal fiber orientations.

		Optimal Stiffness from Simulations (N/mm)	Measured Stiffness from Experiments (N/mm)
$\alpha = 10$ $f = 0.1$	k_y	2.47	2.29 ± 0.11
	k_x	13.44	13.53 ± 0.57
$\alpha = 40$ $f = 0.1$	k_y	11.72	8.08 ± 0.06
	k_x	51.31	55.58 ± 0.13

Table S1 Stiffness values from simulations and experiments (averaged) for the 2D structures optimized with setup from Case 3 and with $\alpha = 10$ (three structures of size: 300×200 mm, 210×120 mm and 120×80 mm) and 40 (one structure of size 300×200 mm). k_y = stiffness in bending and k_x = stiffness under tension. Measurements for each loading case on each structure were repeated at least 5 times.

	Bending, k_y (N/mm)	Tension, k_x (N/mm)
300×200 mm	2.19 ± 0.02	12.82 ± 0.06
210×120 mm	2.35 ± 0.01	13.72 ± 0.08
120×80 mm	2.44 ± 0.04	14.12 ± 0.09

Table S2 Stiffness values obtained from experiments (averaged) for the 2D beams with $\alpha = 10$ (Case 3) and different sizes: 300×200 mm, 210×120 mm and 120×80 mm.

4.2 Optimal design of 3D structures

	Case 1 (2-fold symmetry)	Case 2 (4-fold symmetry)	Case 3 (Mixed)
Macrostructure Symmetry	(0,0,1) & (0,1,0)	(0,0,1), (0,1,0) & (0,1,1)	(0,0,1), (0,1,0) & (0,1,1)
Microstructure Symmetry	(0,0,1) & (0,1,0)	(0,0,1), (0,1,0), & (0,1,1)	(0,0,1) & (0,1,0)
Symmetry planes schematic	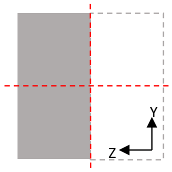	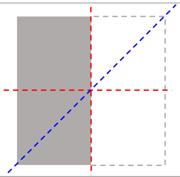	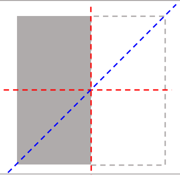
Optimal Design	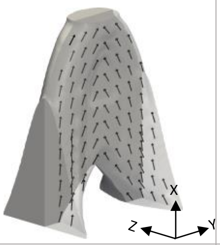	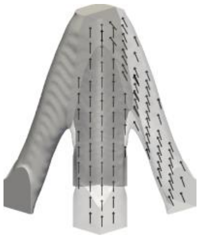	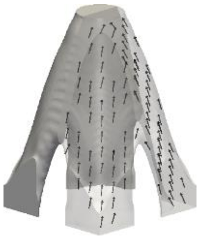
Optimized Stiffness (N/mm)	k_y = 5.43	3.93	4.41
	k_z = 0.72	3.93	3.21
	k_x = 26.72	31.93	27.67

Table S3 Symmetry conditions, optimal design and optimal stiffness values for the three different combinations of symmetry conditions simulated. The symmetry planes are specified in terms of their normals. xy- and xz-planes are shown in red in the schematics while the diagonal plane is shown in blue. k_y and k_z are stiffness values under bending loads along y- and z-directions respectively while k_x is stiffness under tension with load along x-axis.

We used regular 8-noded linear hexahedral elements of size, $h_e = 1.6$ mm to discretize the design domain and a linear filter of radius $6h_e$ for all the 3D problems discussed in the main text. In Figs. 4b-c from the main text, we showed the results of the optimization with different symmetry conditions for the macroscale topology and the microstructure. Table S3 shows the setup used for the three different cases (2-, 4-fold symmetry and the mixed case) and the results obtained. For Case 1 which is 2-fold symmetry (same as the result in Fig. 4b), we set the optimization variables such that the design variables satisfy the following conditions:

$$\begin{gathered}\rho(x,y,z) = \rho(x,y,-z) = \rho(x,-y,z) \\ \theta_i(x,y,z) = \theta_i(x,y,-z) = -\theta_i(x,-y,z) \\ \theta_o(x,y,z) = -\theta_o(x,y,-z) = \theta_o(x,-y,z)\end{gathered} \tag{S13}$$

These conditions mean that the designs are constricted to be symmetrical about *xy*- and *xz*-planes. Similarly, to enforce 4-fold symmetry for Case 2 (same as the result in Fig. 4c), in addition to the conditions stated above we have the following conditions that enforce designs to be symmetric about the plane bisecting *xy*- and *xz*-planes:

$$\begin{aligned} \rho(x,y,z) &= \rho(x,z,y) \\ \theta_i(x,y,z) &= -\theta_i(x,z,y) \\ \theta_o(x,y,z) &= \theta_o(x,z,y) \end{aligned} \tag{S14}$$

For Case 3 (same as the result in Fig. 4d), just as before we use eq. (S13) to enforce symmetry about *xy*- and *xz*-planes for ρ, θ_i and θ_o. In addition, we enforce the condition: $\rho(x,y,z) = \rho(x,z,y)$ which means 4-fold symmetry just as in Case 2 for the macrostructure but only 2-fold symmetry for microstructure as in Case 1. The enforced symmetries become evident in the measured mechanical response shown in Table S3.

Fig. S14 shows the convergence history for the results shown in Figs. 4b-e in the main text (includes Cases 1-3 shown in Table S3). Table S4 tabulates the stiffness values obtained from the simulations and the experiments for various loading directions for the results shown in Figs. 4d and 4e of the main text with the same $f = 0.1$ but different α (=10 and 40 respectively) where the formulation in Case 3 is used. Fig. S15 and Fig. S16 show the $\rho = 0.5$ iso-contour surface for different design iterations for the same. The results are very similar to the 2D case; we show one half of the structure opaque and the other half transparent with fiber orientations for visualization purposes. Fig. S17 shows the cross-sections at different locations along the length of these beams.

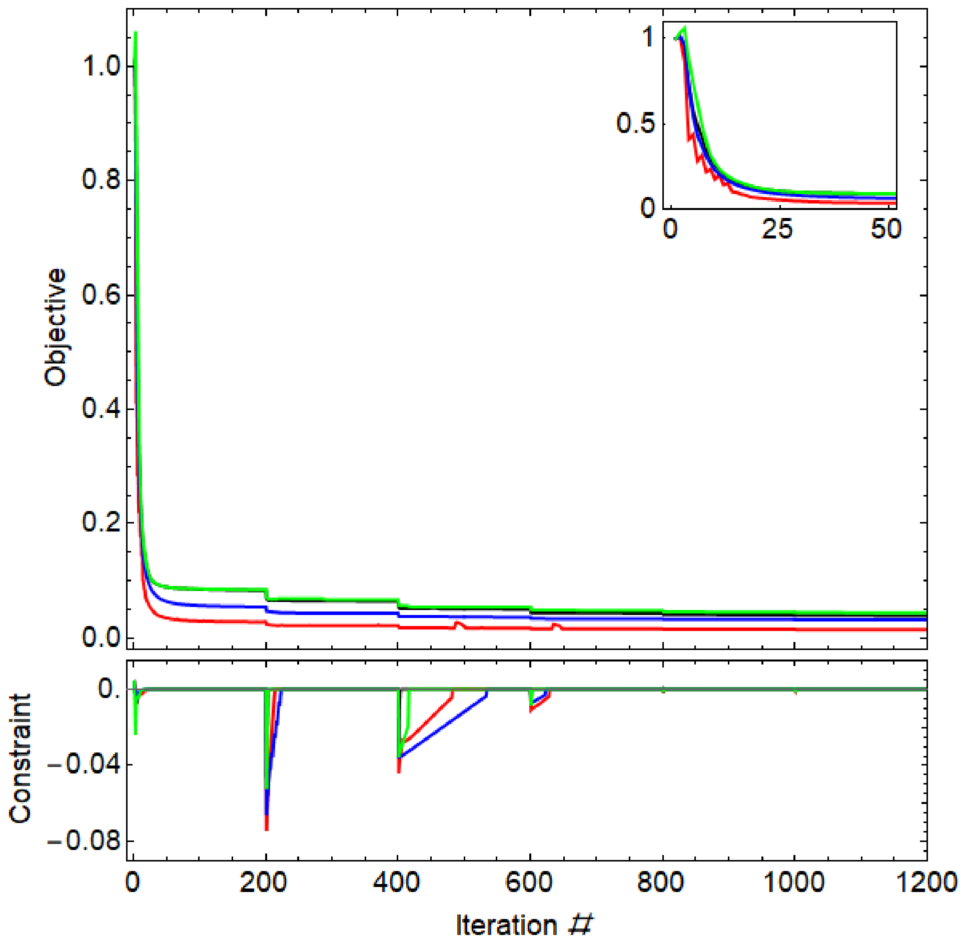

Fig. S14 Convergence history of the objective (top) and constraint (bottom) for the results shown in Figs. 4b, 4c, 4d and 4e. Inset shows the first 50 iterations of the objective. Blue curve - Case 1 in Table S3/result in Fig. 4b, Green Curve – Case 2 in Table S3/result in Fig. 4c, Black curve – Case 3 in Table S3/result in Fig. 4d with $\alpha = 10$ *and Red – same as Case 3 but with* $\alpha = 40$*/result in Fig. 4e.*

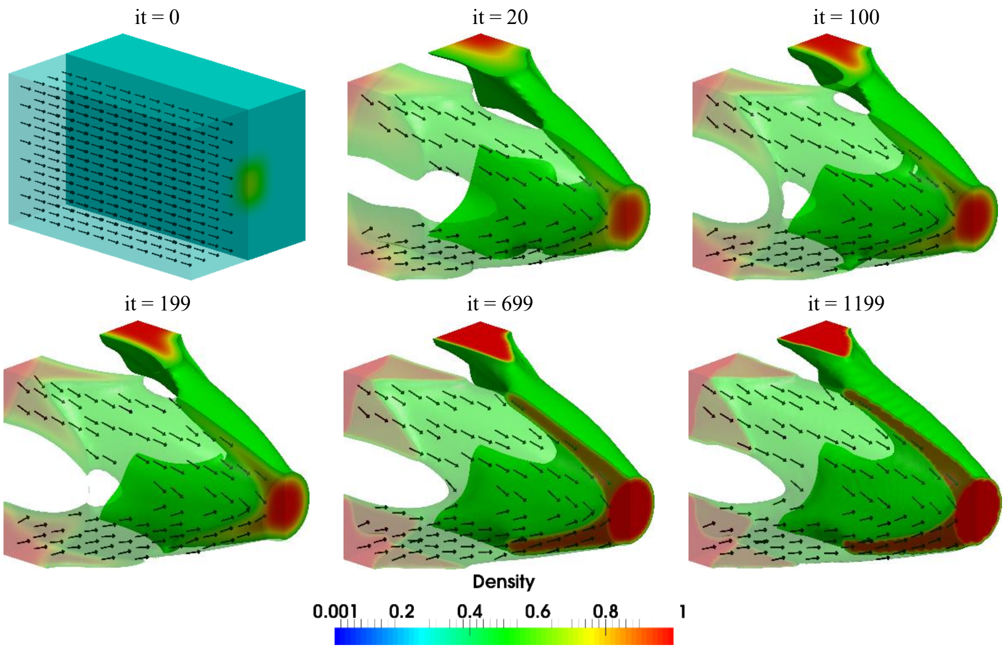

Fig. S15 Designs at different iterations for Case 3 with α = 10 (Fig. 4d in main text). The fiber orientations are also shown here.

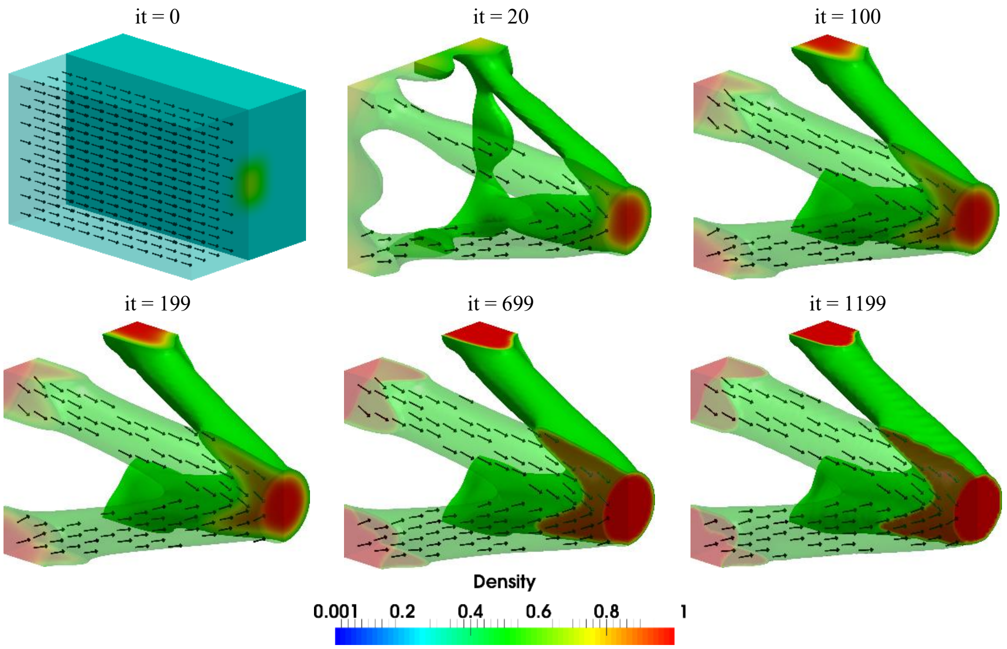

Fig. S16 Designs at different iterations for Case 3 with α = 40 (Fig. 4e in main text). The fiber orientations are also shown here.

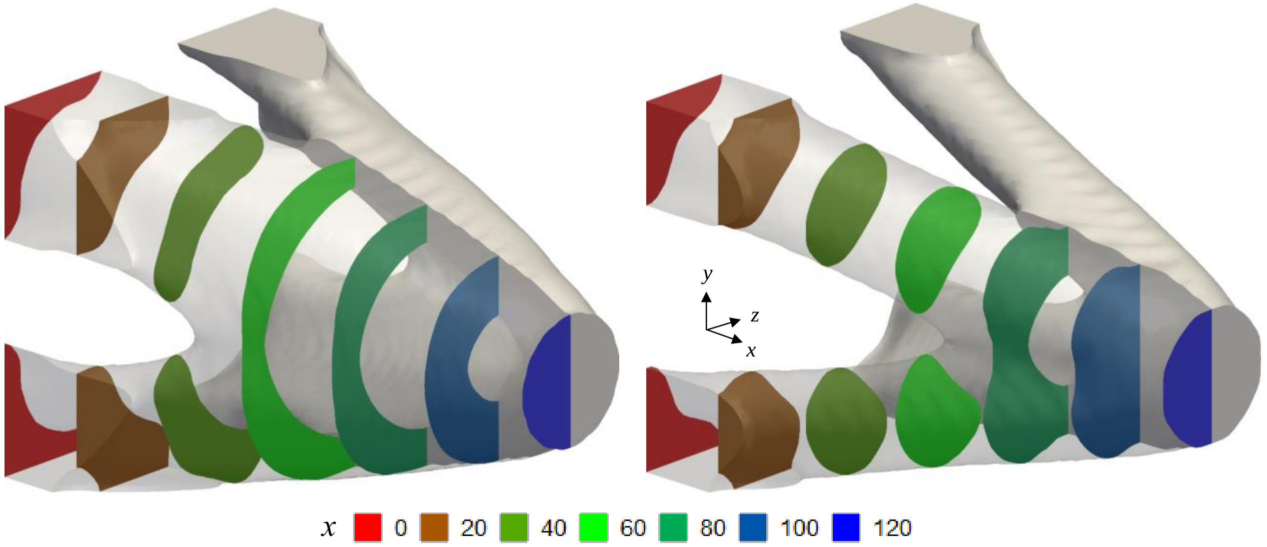

Fig. S17 The cross-section of the 3D structures designed with the mixed symmetry conditions (Case 3) at different locations along the x-axis - $\alpha = 10$ *case on the left and* $\alpha = 40$ *on the right*

		Optimal Stiffness from Simulations (N/mm)	Measured Stiffness from Experiments (N/mm)
$\alpha = 10$ $f = 0.1$	k_y	4.41	4.15 ± 0.04
	k_z	3.21	2.58 ± 0.03
	k_x	27.67	25.58 ± 0.33
$\alpha = 40$ $f = 0.1$	k_y	18.87	15.86 ± 0.21
	k_z	9.24	7.61 ± 0.06
	k_x	90.33	105.41 ± 1.53

Table S4 Stiffness values from simulations and experiments (averaged) for the two 3D beam structures designed with the mixed symmetry conditions (Case 3) and with $\alpha =$ *10 and 40 respectively under different loading conditions.* k_y *and* k_z *are stiffness values under bending loads along y- and z-directions respectively while* k_x *is stiffness under tension with load along x-axis. Measurements for each loading case on each structure were repeated 6 times.*

5. Experimental Details

5.1 Material Characterization

For the experimental results shown in Fig. 1b in the main text, we printed prismatic bar samples of length 80 mm and a rectangular cross-section with dimensions 20.6x19.83 mm. Samples were printed with fiber aspect ratios α varying from 5-40, while holding the volume fraction, f, and orientation angles θ_i and θ_o fixed at 0.1, 0° and 0° respectively. As a result, the samples had the fibers aligned with the loading direction

(x-) and we then measured the modulus along the fiber axes ($E_x = (\mathbf{C}^{-1})_{11}{}^{-1}$). The samples were 3D printed with their long dimension (x-) aligned with the x- direction of the print tray, i.e., the direction in which the printhead moves when dispensing ink and curing the printed materials. We used the soft elastomer *Tango+* for the matrix and stiff glassy polymer *VeroClear* to print the fibers; both materials are supplied by Stratasys Ltd in resin form. The samples were subjected to uniaxial tension/compression up to 1% strain at 1%/min strain rate. All the experiments were done with Instron 5943 mechanical testing system with a 1 kN load cell. The results are tabulated in Table S5. We used the average of the moduli obtained from tension and compression to fit the experimental results to the Mori-Tanaka homogenization theory with E_m and E_f as the fitting parameters. We obtained the best fit with E_m = 1.22 MPa and E_f = 1030.23 MPa. However, the moduli obtained for these materials when measured individually are about 0.75 MPa and 1.1 GPa which are in line with other independent measurements for the same or closely related materials.[17–19] The range of values provided by the supplier, Stratasys for *VeroClear* are 2-3 GPa.[20]

Aspect Ratio (α)	Tension (MPa)	Compression (MPa)
5	1.84	1.86
10	3.65	3.75
20	9.57	9.72
30	16.71	16.95
40	24.42	23.69
∞	100.1	110.32

Table S5 Measured uniaxial tension and compression moduli (E_x) for composite bars used for material characterization with $\theta_i = \theta_o = 0$ and $f = 0.1$.

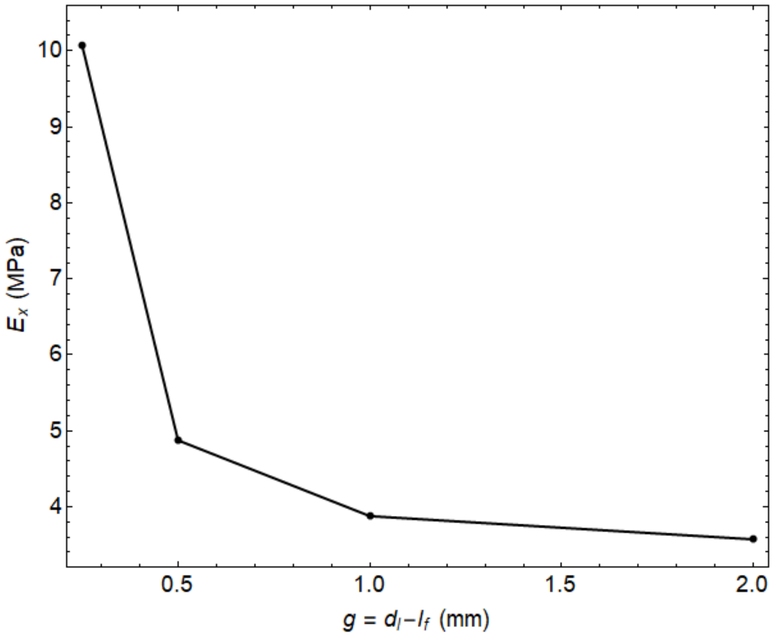

Fig. S18 Measured tension moduli, E_x for composite bars with different initial gaps, $g = d_l - l_f$ but same θ_i = θ_o = 0, α = 10 and f = 0.1.

The discrepancy between the measured values and the fitted values for the elastomeric matrix can be explained by *smearing* of the interface between the fiber and matrix materials. This smearing, we surmise, is the result of mixing of the matrix and fiber materials during the printing process which manifests as an increased effective matrix modulus. Fig. S18 shows the variation of the effective tension modulus, E_x of composite bars with $g = d_a - l_f$, the initial gap along the length of the fibers during the microstructure realization step while the microstructural descriptors are all kept the same (θ_i = θ_o = 0, α = *10* and f = 0.1). We noticed that the smearing effect can only be minimized but not avoided entirely and choose g to be at least 1 mm.

5.2 Validation of Optimal Structures

	Tension	Bending
2D Beam	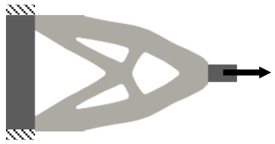	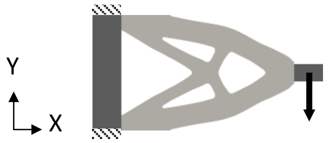
3D Beam	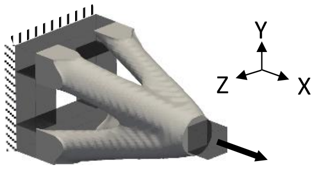	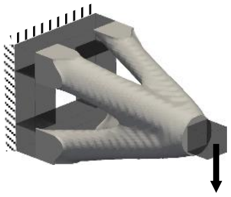 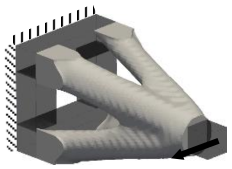

Table S6 Schematics showing the setups used for experimental validation of the 2D and 3D optimal beam structures with the arrows indicating the direction of the applied load. The black transparent structures shown here aid in clamping and loading the structures.

We also printed the optimal beam structures along the print tray's x-direction and then subjected them to mechanical testing to validate the simulation results. We show through a table of schematics in Table S6, the experimental setups used under different loading scenarios viz. tension and bending. We took advantage of the multi-material printing capabilities to print stiff supporting structures made of the Vero material – a block at the base of the beam to assist in clamping the structure and a short tab at the loading end (both shown in black in the schematics in Table S6). We fixed the base structure in a rigid frame in an appropriate orientation (vertically for tension and horizontally for bending) thus clamping the base of the beam while the end tab was attached to the loading fixture of the mechanical testing machine so that a desired load can be transmitted to the beam.

The comparison between experimental and simulation results are presented in Table S1 and Table S4 for 2D and 3D structures respectively. The discrepancies between them might result from the smearing effect discussed in the previous sub-section. The material compilation process introduces a small degree of non-uniformity as evidenced in Fig. S7 leading to non-uniform spacing between the fibers which in turn leads to slight deviations from the expected behavior. In addition, the discrete nature of the voxel-based printing process leading to inaccurate representation of the fibers and printing flaws might also be contributing to the discrepancies. The inherent anisotropy induced by the Connex3 Objet500 printer as reported in the literature[19,21] might also be a contributing factor which we haven't considered in this study.

6. References

1. Mori, T. & Tanaka, K. Average stress in matrix and average elastic energy of materials with misfitting inclusions. *Acta Metall.* **21,** 571–574 (1973).
2. Benveniste, Y. A new approach to the application of Mori-Tanaka's theory in composite materials. *Mech. Mater.* **6,** 147–157 (1987).
3. Dunn, M. L., Ledbetter, H., Heyliger, P. R. & Choi, C. S. Elastic constants of textured short-fiber composites. *J. Mech. Phys. Solids* **44,** 1509–1541 (1996).
4. Dunn, M. L., Taya, M., Hatta, H., Takei, T. & Nakajima, Y. Thermal Conductivity of Hybrid Short Fiber Composites. *J. Compos. Mater.* **27,** 1493–1519 (1993).
5. Dunn, M. L. & Ledbetter, H. Elastic-plastic behavior of textured short-fiber composites. *Acta Mater.* **45,** 3327–3340 (1997).
6. Ogi, H. *et al.* Elastic constants and internal friction of an SiC-fiber-reinforced Ti-alloy-matrix crossply composite: measurement and theory. *Acta Mater.* **47,** 2787–2796 (1999).
7. Tucker III, C. L. & Liang, E. Stiffness predictions for unidirectional short-fiber composites: Review and evaluation. *Compos. Sci. Technol.* **59,** 655–671 (1999).
8. Eshelby, J. D. The Determination of the Elastic Field of an Ellipsoidal Inclusion, and Related Problems. *Proc. R. Soc. A Math. Phys. Eng. Sci.* **241,** 376–396 (1957).
9. Mura, T. *Micromechanics of defects in solids*. (Springer Netherlands, 1982). doi:10.1007/978-94-011-9306-1
10. Guest, J. K., Prévost, J. H. & Belytschko, T. Achieving minimum length scale in topology optimization using nodal design variables and projection functions. *Int. J. Numer. Methods Eng.* **61,** 238–254 (2004).
11. Sigmund, O. Morphology-based black and white filters for topology optimization. *Struct. Multidiscip. Optim.* **33,** 401–424 (2007).
12. Wang, F., Lazarov, B. S. & Sigmund, O. On projection methods, convergence and robust formulations in topology optimization. *Struct. Multidiscip. Optim.* **43,** 767–784 (2011).
13. Svanberg, K. A Class of Globally Convergent Optimization Methods Based on Conservative Convex Separable Approximations. *SIAM J. Optim.* **12,** 555–573 (2002).
14. Ketchel, J. S. & Larochelle, P. M. Collision Detection of Cylindrical Rigid Bodies Using Line Geometry. in *Volume 7: 29th Mechanisms and Robotics Conference, Parts A and B* **2005,** 811–825 (ASME, 2005).
15. Panagiotis, M. & Payne, A. O. Working with multi-scale material distributions. in *ACADIA 2013 Adapative Architecture: Proceedings of the 33rd Annual Conference of the Association for Computer Aided Design in Architecture* 43–50 (2013).
16. Panagiotis, M. & Payne, A. O. Monolith: A Voxel-Based Modeling Engine For Multimaterial 3D

Printing. (2015). Available at: http://www.monolith.zone/. (Accessed: 17th August 2017)
17. Schittny, R., Bückmann, T., Kadic, M. & Wegener, M. Elastic measurements on macroscopic three-dimensional pentamode metamaterials. *Appl. Phys. Lett.* **103,** 231905 (2013).
18. Shen, J., Zhou, S., Huang, X. & Xie, Y. M. Simple cubic three-dimensional auxetic metamaterials. *Phys. status solidi* **251,** 1515–1522 (2014).
19. Dalaq, A. S., Abueidda, D. W. & Abu Al-Rub, R. K. Mechanical properties of 3D printed interpenetrating phase composites with novel architectured 3D solid-sheet reinforcements. *Compos. Part A Appl. Sci. Manuf.* **84,** 266–280 (2016).
20. PolyJet Materials. Available at: http://www.stratasys.com/materials/material-safety-data-sheets/polyjet/transparent-materials. (Accessed: 17th August 2017)
21. Mueller, J. & Shea, K. The effect of build orientation on the mechanical properties in inkjet 3D-printing. in *International Solid Freeform Fabrication Symposium, Austin, TX, United States* (2015).
